# Propanol production through microbial fermentation of biomass

**DOI:** 10.1186/s40643-025-00966-6

**Published:** 2025-11-05

**Authors:** Caiping Sun, Luxin Yang, Chuyun Zhao, Huan Li, Zhou Deng, Guangli Liu, Chunxu Wu

**Affiliations:** 1 Tsinghua Shenzhen International Graduate School, Tsinghua University, Shenzhen, 518055 China; 2 Shenzhen Lisai Environmental Protection Co. Ltd.,, Shenzhen, 518055 China; 3 School of Environmental Science and Engineering, Sun Yat-Sen University, Guangzhou, 510006 China; 4 Shenzhen Qingzhi Environmental Protection Co. Ltd.,, Shenzhen, 518055 China

**Keywords:** Biosynthesis, Fermentation, Metabolic engineering, Propanol

## Abstract

As a key short-chain alcohol compound, propanol has a diverse range of applications in solvents, pharmaceutical intermediates, fuel additives, and other fields. With the increasing global demand for sustainable development and green chemistry, the production technology of biopropanol is gradually shifting from traditional petroleum-based chemical synthesis to biosynthesis based on microbial fermentation. This paper reviews the recent research progress in the field of biopropanol production, encompassing various aspects such as natural propanol-producing strains, genetically engineered strains, metabolic pathway design, fermentation process optimization, and downstream purification technologies. Despite the remarkable progress in biopropanol production technology, it still faces numerous challenges, including the low production efficiency of natural microorganisms, the strong inhibitory effect of the product, and poor substrate conversion rates. Future research can be directed toward optimizing fermentation conditions, integrating downstream separation technology, and developing highly active key enzyme components and artificial metabolic pathways to enhance the production efficiency of biopropanol and improve its feasibility for industrial applications.

**Graphical abstract:**

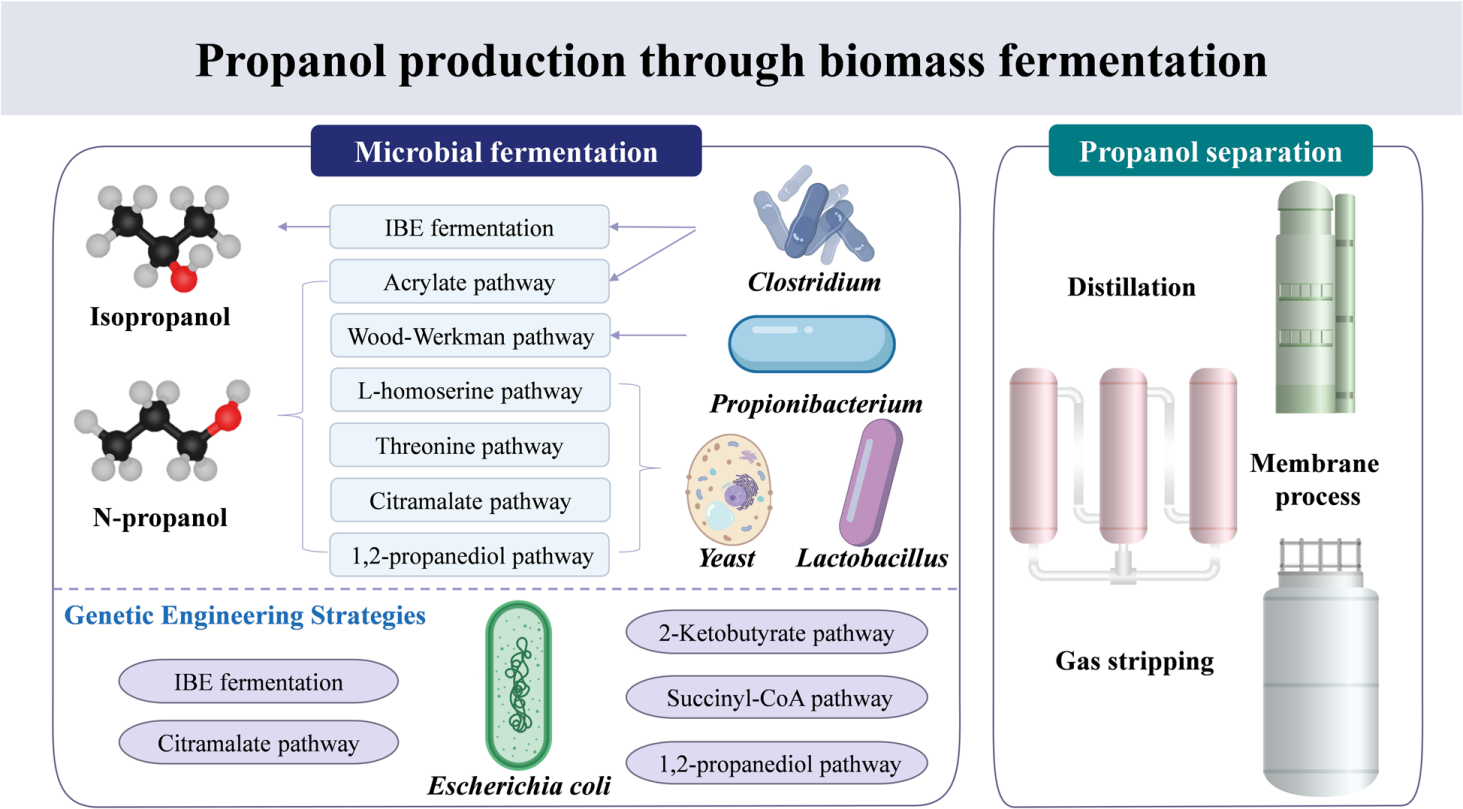

## Introduction

Propanol (C_3_H_7_OH) is a significant short-chain alcohol compound containing two isomers, n-propanol (1-propanol) and isopropanol (2-propanol). Propanol is a key intermediate in the synthesis of organic compounds such as propylene acetate and acrylic acid. It is an essential raw material in the chemical industry and has extensive applications across various sectors, including coatings, pharmaceuticals, pesticides, and feed additives (Abrar et al. 2023). With the acceleration of global industrialization, the demand for propanol in the market has grown in recent years. According to statistics, the global propanol market reached USD 3.76 billion in 2023 and is expected to expand steadily at a compound annual growth rate (CAGR) of 6.6% between 2023 and 2030. Currently, n-propanol is primarily produced through the carbonyl synthesis of propanal from ethylene, which is then catalytically hydrogenated (Kuhz et al. 2017). Isopropanol is primarily industrially produced through the hydration of propylene or the hydrogenation of acetone (Dubois et al. 2022). However, these traditional chemical synthesis methods result in high energy consumption and carbon emissions, making it challenging to meet the current requirements of green chemistry and sustainable development.

Propanol is also a potential green fuel or fuel materials. Bioalcohol fuels (e.g., biomethanol, bioethanol, biopropanol) are considered an alternative to fossil fuels due to their renewability and environmental friendliness, which benefits energy security and environmental protection. Ethanol is currently the most widely used alcohol fuel with a high energy density (26.7 MJ/kg) (Nanda et al. 2020), facilitating long-distance transportation (Ramsey et al. 2023). Methanol (19.9 MJ/kg) exhibits competitiveness in navigation due to its diverse synthesis pathways (e.g., carbon dioxide hydrogenation or biomass gasification) and relatively lower production costs (Kuhz et al. 2017; Zhou et al. 2024). Green alcohol can be used to synthesis fully biobased diesel together with waste oil through transesterification, and also used to manufacture sustainable aviation fuel (SAF) through the Alcohol-to-Jet pathway. Despite of the rapid growth of electric transportation in China, the global liquid biofuel demand grow continuously from 5.0 EJ in 2024 to 6.2 EJ in 2030 according to the newest report of the International Energy Agency (IEA 2025). Compared with methanol and ethanol, propanol exhibits unique advantages, including a high calorific value (30.6 MJ/kg) and a high octane number (118), leading to efficient and stable energy release (Nanda et al. 2020). Additionally, its oxygen content enhances combustion efficiency while reducing harmful emissions such as CO and NO_x_. Experimental studies have shown that biopropanol effectively reduces unburned hydrocarbon emissions when used as a single fuel or blended with gasoline/diesel (Zhou et al. 2019).

Biopropanol synthesis mainly relies on biomass gasification and microbial fermentation (Kumar Sarangi et al. 2023; Schubert 2020). The gasification method converts biomass into syngas rich in carbon monoxide and hydrogen through high-temperature and anoxic conditions, followed by a catalytic reaction to synthesize propanol. This method has the advantages of a wide source of feedstock and large-scale production (Daniell et al. 2012). However, it suffers from high energy consumption in the gasification process, harsh reaction conditions, and poor catalyst stability (Marie-Rose et al. 2015). Fermentation refers to the metabolic conversion of renewable biomass resources using microorganisms (e.g., *Clostridium*) to produce propanol, which offers the advantages of mild reaction conditions and environmental friendliness, and has become a focus of current research (Ávila et al. 2023; Ko et al. 2022b). Natural strains have a low yield of propanol, whereas some microorganisms have been engineered into strains with a high propanol synthesis capacity (Jojima et al. 2008). However, to abandon expensive substrates or food crops (Liu et al. 2019a; Thi Quynh Le and Lee, 2022), the current research focuses on propanol production using low-cost and non-food-based carbon sources, especially lignocellulose (dos Santos Vieira et al. 2019), biodiesel by-products (Shi et al. 2022), and food waste (Procentese et al. 2018). Engineering strains are not robust when dealing with complex biomass wastes (Zhou et al. 2019).

In summary, the microbial fermentation method shows great potential in achieving green and sustainable propanol production. However, its industrial application still faces several challenges, including the screening and targeted modification of efficient strains, enhancing tolerance to toxicity in the fermentation process, and optimizing downstream extraction and purification processes. Therefore, in this paper, the latest research progresses of the currently used microbial strains and their metabolic engineering strategies, the construction and regulation mechanisms of biosynthetic pathways, as well as the downstream isolation and purification methods are reviewed around the microbial synthesis system of biopropanol, aiming at providing theoretical foundations and technological supports to realize the efficient production of biopropanol, as well as giving the reference direction for the subsequent industrial development.

## Microbial fermentation

### Natural propanol-producing strains

Under natural conditions, certain microorganisms can synthesize propanol through metabolic pathways, primarily by fermenting organic substrates such as glucose. These microorganisms typically employ reductive metabolic pathways to generate energy while converting carbon sources into various fermentation products, including propanol. Numerous microorganisms capable of naturally producing propanol have been identified to date (Table 1), including species belonging to the genera *Clostridium*, *Propionibacterium*, *Lactobacillus*, and *Saccharomyces*. These microorganisms exhibit substantial differences in their mechanisms of propanol biosynthesis, production yields, and substrate utilization pathways, highlighting their value as model systems for exploring the natural biotechnological potential of propanol production.

**Table 1:** Biopropanol production by different natural microorganisms

Strains	Cultivation conditions	Propanol concentration (mmol/L)	Yield (g/g)	Productivity [g/(L h)]	References
Naturally propanol-producing strains					
*C. beijerinckii* VPI 2968	20 g/L glucose; pH = 6.8, T = 35 ℃	44.8	–	–	(George et al. 1983)
*C. beijerinckii* VPI 2982	20 g/L glucose; pH = 6.8, T = 35 ℃	41.3	–	–	(George et al. 1983)
*C. butylicum* NRRL B-593	20 g/L glucose; pH = 6.8, T = 35 ℃	61.7	–	–	(George et al. 1983)
*C. aurantibutyricum* ATCC 17777	20 g/L glucose; pH = 6.8, T = 35 ℃	4.5	–	–	(George et al. 1983)
*C. acetobutylicum* VPI 2676	20 g/L glucose; pH = 6.8, T = 35 ℃	3.3	–	–	(George et al. 1983)
*C. beijerinckii optinoii*	28.34 g/L glucose; T = 36 ℃	57.4	–	–	(Moon et al. 2015)
*C. beijerinckii optinoii*	29.47 g/L glucose and molasses; T = 36 ℃	79.4	–	–	(Moon et al. 2015)
*C. beijerinckii optinoii*	32.05 g/L sugarcane molasses; T = 36 ℃	73.2	–	–	(Moon et al. 2015)
co-culturing *C. beijerinckii* with* C. tyrobutyricum*	52.02 g/L cassava bagasse hydrolysate; pH = 6.0, T = 36 ℃	126.9	–	–	(Zhang et al. 2016)
*C. beijerinckii* BGS1	60 g/L glucose; pH = 4.5 ~ 6.5, T = 35 ℃	56.7	–	–	(Zhang et al. 2018)
*C. beijerinckii* BGS1	60 g/L sucrose; pH = 4.5 ~ 6.5, T = 35 ℃	42.8	–	–	(Zhang et al. 2018)
*C. beijerinckii* DSM 6423	21 g/L coffee silverskin hydrolysate; T = 37 ℃	36.6	0.16	0.055	(Procentese et al. 2018)
*C. beijerinckii* DSM6423	44.1 g/L tomato waste; pH = 6.0, T = 35 ℃	121.5	–	–	(Hijosa-Valsero et al. 2019)
*C. beijerinckii* DSM6423	60 g/L glucose; T = 35 ℃	48.3	0.09	0.06	(Rochón et al. 2019)
*C. beijerinckii* DSM6423	60 g/L sucrose; T = 35 ℃	30.0	0.09	0.03	(Rochón et al. 2019)
*C. beijerinckii* DSM6423	60 g/L sugarcane-sweet sorghum juice; T = 35 ℃	63.2	0.11	0.08	(Rochón et al. 2019)
*C. beijerinckii* DSM6423	60 g/L sugarcane juice; T = 35 ℃	30.0	0.11	0.09	(Rochón et al. 2019)
*C. acetobutylicum DSM 792*	44.1 g/L tomato waste; pH = 6.0, T = 35 ℃	137.8	–	–	(Hijosa-Valsero et al. 2019)
*C. beijerinckii* C-01	24 g/L rice straw hydrolysate; pH = 6.5, T = 35 ℃	33.3	–	–	(Dalal et al. 2019)
*C. beijerinckii* BGS1	90 g/L glucose; pH = 6.0, T = 37 ℃	48.3	–	–	(Cui et al. 2020)
*C. beijerinckii* DSM 6423	32.1 g/L sugarcane bagasse hydrolysates; pH = 6.0 ~ 6.2, T = 35 ℃	28.3	–	–	(Vieira et al. 2021)
*C. beijerinckii DSM 6423*	60 g/L glucose; T = 35 ℃	64.2	0.11	–	(Ferreira dos Santos Vieira et al. 2022)
*C. beijerinckii* XH29	60 g/L glucose and acetone; T = 35 ℃	113.1	–	–	(Yao et al. 2022)
*P. acidipropionici* ATCC 25562	20 g/L glycerol; pH = 6.8, T = 30 ℃	35.9	0.036	0.0027	(Barbirato et al. 1997)
*P. acidipropionici* ATCC 25562	20 g/L glycerol; pH = 7.0, T = 30 ℃	–	0.13	0.42	(Himmi et al. 2000)
*P. acidipropionici* ATCC 25562	20 g/L glucose; pH = 7.0, T = 30 ℃	–	0.04	0.24	(Himmi et al. 2000)
*P. freudenreichii ssp. shermanii* ATCC 9614	20 g/L glycerol; pH = 7.0, T = 30 ℃	–	0.09	0.18	(Himmi et al. 2000)
*P. freudenreichii ssp. shermanii* ATCC 9614	20 g/L glucose; pH = 7.0, T = 30 ℃	–	0.06	0.07	(Himmi et al. 2000)
*P. acidipropionici* ATCC4965	40 g/L glycerol; pH = 6.5, T = 30 ℃	17.0	–	–	(Liu et al. 2011)
*P. acidipropionici* DSM 4900	40 g/L glycerol; pH = 6.5, T = 32 ℃	36.3	–	–	(Dishisha et al. 2012)
*S. cerevisiae*	95 g/L agave tequilana weber; T = 35 ℃	0.5	0.032	0.0043	(Díaz-Montaño et al. 2008)
*Kloeckera africana*	95 g/L agave tequilana weber; T = 35 ℃	0.3	0.022	0.0029	(Díaz-Montaño et al. 2008)
*Kloeckera apiculata*	95 g/L agave tequilana weber; T = 35 ℃	0.2	0.014	0.0013	(Díaz-Montaño et al. 2008)
*L. delbrueckii* H1 and *S. cerevisiae*	sorghum malt mash; T = 24 ℃	2.2	−	–	(Djameh et al. 2019)

#### Clostridium

**Fig. 1:**
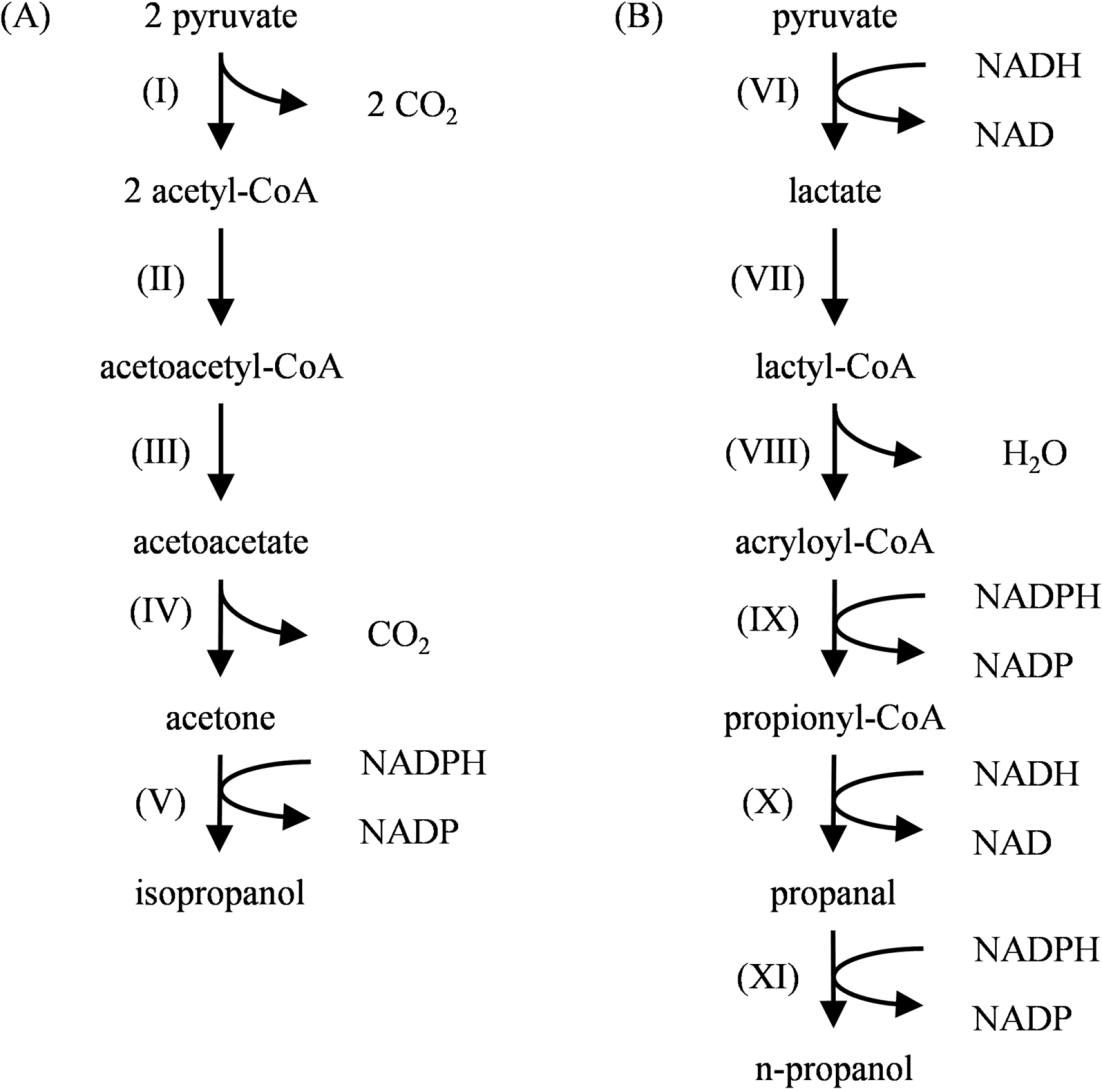
Natural propanol synthesis pathways in *Clostridium* (Walther and François 2016). **A** IBE fermentation pathway for isopropanol synthesis; **B** Acrylate pathway for n-propanol synthesis. (Ⅰ) Pyruvate-Ferredoxin Oxidoreductase; (Ⅱ) Acetoacetyl-CoA thiolase; (Ⅲ) Acetoacetyl-CoA: acetate CoA-transferase; (Ⅳ) Acetoacetate decarboxylase; (Ⅴ) Isopropanol dehydrogenase; (Ⅵ) Lactate dehydrogenase; (Ⅶ) Propionyl-CoA:lactate CoA-transferase; (Ⅷ) Lactyl-CoA dehydratase; (Ⅸ) Acryloyl-CoA reductase; (Ⅹ) Propionaldehyde dehydrogenase; (Ⅺ) Propanol dehydrogenase

*Clostridium* is a group of Gram-positive and strictly anaerobic bacteria capable of producing spores, and it can synthesize propanol through multiple metabolic pathways. Among these, *C. beijerinckii* and its related strains can synthesize various organic solvents through the acetone-butanol-ethanol (ABE) fermentation pathway, one of the most essential industrial fermentation technologies in the early twentieth century. Some *Clostridium* can alter their metabolic pathways to reduce acetone into isopropanol during ABE fermentation, forming the isopropanol-butanol-ethanol (IBE) fermentation model, also known as the acetone-dependent isopropanol pathway (Hocq and Sauer 2022). As illustrated in Fig. 1A, one molecule of glucose undergoes glycolysis to generate two molecules of pyruvate, which are subsequently converted into two molecules of acetyl-CoA under the catalysis of pyruvate-ferredoxin oxidoreductase (PFOR), releasing two molecules of CO_2_. The acetyl-CoA molecules then condense to form acetoacetyl-CoA via acetoacetyl-CoA:acetate CoA-transferase, followed by hydrolysis and decarboxylation to produce acetone. Finally, acetone is reduced to isopropanol (Ko et al. 2022b; Tamakawa et al. 2013; Walther and François 2016). When the carbon flux is fully directed toward acetone synthesis, the theoretical maximum yield of isopropanol reaches 0.5 mol/mol glucose. However, in actual fermentation processes, due to the low expression activity of NADPH-dependent primary/secondary alcohol dehydrogenase (Xin et al. 2017), the actual yield of isopropanol is significantly lower than the theoretical value (George et al. 1983).

In addition to the acetone-dependent isopropanol pathway, some *Clostridium* strains can synthesize n-propanol. Strains such as *C. propionicum* can synthesize trace amounts of n-propanol via the acrylate pathway, which uses lactate as a precursor (Fig. 1B). Pyruvate undergoes a series of enzymatic conversions catalyzed by lactate dehydrogenase, lactoyl-CoA dehydratase, and acryloyl-CoA reductase, forming lactoyl-CoA, acryloyl-CoA, and propionyl-CoA, which is further converted into propionic acid (Le, 2021; Reichardt et al. 2014). Under specific conditions, propionyl-CoA can be sequentially reduced by acylating propionaldehyde dehydrogenase and propanol dehydrogenase to form propanal and, subsequently, n-propanol (Walther and François 2016). *C. neopropionicum* X4 strain can produce n-propanol through the acrylate pathway under anaerobic fermentation with 30 mmol/L ethanol, reaching a concentration of 1.52 mmol/L (Tholozan et al. 1992). Additionally, when *C. acetobutylicum* produces solvents (such as ethyl acetate, butanol, and n-propanol) under neutral pH conditions, adding a small amount of propionic acid at pH levels of 5.0 or 7.0 can promote the synthesis of n-propanol (Holt et al. 1984). This result suggests that propionic acid may be converted into n-propanol through a metabolic pathway.

Currently, fermentation studies on propanol production primarily focus on *C. beijerinckii*. This fermentation process is highly dependent on culture conditions, including inoculum, carbon source (Zhang et al. 2018), fermentation temperature (Ferreira dos Santos Vieira et al. 2022), pH (Zhang et al. 2018), and so on. Studies have shown that *Clostridium* can utilize various carbon sources for propanol fermentation, including glucose, xylose, fructose, and biomass waste such as cassava peels, sugarcane bagasse, and coffee husks. In the IBE fermentation, *C. beijerinckii* BGS1 produced 0.04 g isopropanol from 1 g sucrose at 35 °C and pH 5.0–6.5 (Zhang et al. 2018). *C. beijerinckii* DSM 6423 fermentation of coffee silverskin resulted in an isopropanol yield of 0.16 g/g sugar (Procentese et al. 2018). Sequential batch fermentation of cassava-sugarcane hydrolysate at 36 °C and an initial pH of 6.0 yielded isopropanol up to 0.22 g/g sugar (Zhang et al. 2016). Similarly, *C. beijerinckii* DSM 6423 fermentation of glucose at 35 °C resulted in an isopropanol yield of 0.11 g/g glucose (Ferreira dos Santos Vieira et al. 2022).

Microbial co-culture fermentation allows efficient synthesis of bio-based propanol. For example, propionic acid bacteria produce propionic acid precursors via the Wood-Werkman pathway, while *C. beijerinckii* completes efficient conversion via the IBE pathway. When the initial strain ratio (R value) was optimized to 1,000,000, the propanol yield reached 0.24 g/g mixed carbon source (50% sucrose + 50% glycerol) (Hocq and Sauer 2022). Similarly, *C. tyrobutyricum* can enhance sugar utilization efficiency and provide precursors such as acetic acid and butyric acid, which can be further converted into bio-propanol by *C. beijerinckii*, thereby improving overall fermentation efficiency to 0.16 g/g sugar (Moon et al. 2015).

#### Propionibacterium

**Fig. 2:**
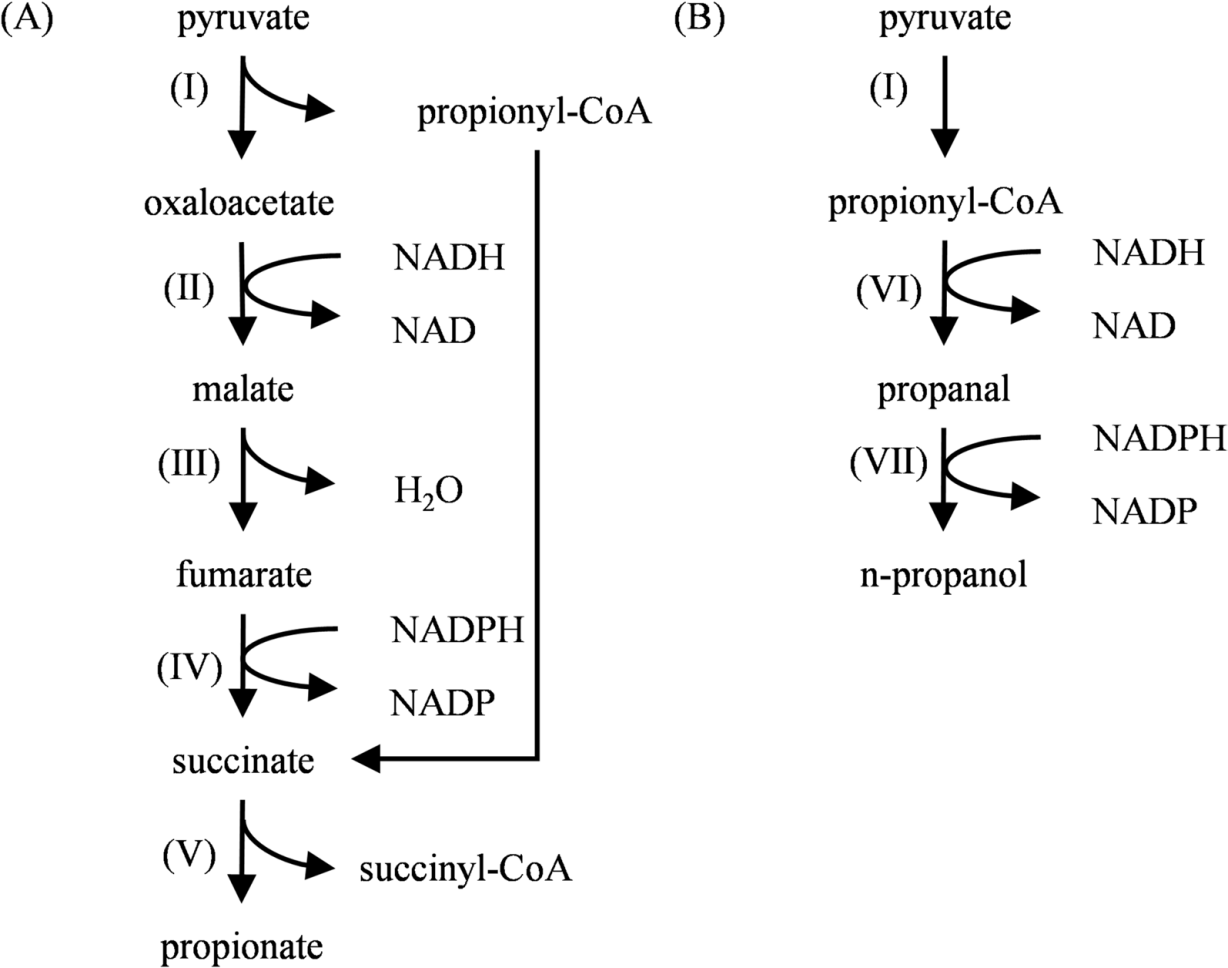
Natural propanol synthesis pathways in *Propionibacterium* (Allen et al. 1964; Wood 1981). **A** Wood-Werkman pathway for propionate synthesis; **B** N-propanol synthesis pathway. (Ⅰ) Methylmalonyl-CoA carboxytransferase; (Ⅱ) Malate dehydrogenase; (Ⅲ) Fumarase; (Ⅳ) Fumarate reductase; (Ⅴ) Propionyl-CoA: succinate CoA-transferase; (Ⅵ) Propionaldehyde dehydrogenase; (Ⅶ) Propanol dehydrogenase

*Propionibacterium* is a group of Gram-positive and facultative anaerobic bacteria (Navone et al. 2018) widely distributed in natural environments. It can convert carbon sources into propionic acid through the Wood-Werkman cycle (the dicarboxylic acid cycle). This cycle begins with pyruvate, which undergoes the following core metabolic steps (Fig. 2A): Under the catalysis of methylmalonyl-CoA carboxyltransferase, one molecule of pyruvate is converted into one molecule of oxaloacetate by receiving a carboxyl group from methylmalonyl-CoA, while releasing one molecule of propionyl-CoA. Oxaloacetate then enters the reductive branch of the tricarboxylic acid (TCA) cycle, sequentially undergoing reductions by malate dehydrogenase, fumarase, and fumarate reductase, forming in sequence one molecule of malate, fumarate, and succinate. Finally, propionyl-CoA:succinate CoA-transferase catalyzes the reaction between succinate and propionyl-CoA, generating one molecule of succinyl-CoA and one molecule of propionic acid (Allen et al. 1964; Wood 1981). Under specific conditions (Fig. 2B), such as highly reducing substrates like glycerol, propionyl-CoA produced in this cycle can be reduced in two steps to generate n-propanol.

However, n-propanol is rarely detected in the natural fermentation of *Propionibacterium*. Currently, only a few reports indicate that *P. freudenreichii* ATCC 9614 (Himmi et al. 2000), *P. acidipropionici* ATCC 25562 (Liu et al. 2011), *P. acidipropionici* ATCC 4965, and *P. acidipropionici* DSM 4900 (Dishisha et al. 2012) can convert glucose or glycerol into 0.5–2.5 g/L of n-propanol. To overcome this limitation, researchers have explored metabolic engineering strategies to enhance n-propanol production. For example, *P. freudenreichii* was engineered by introducing a bifunctional aldehyde/alcohol dehydrogenase (*adhE*) gene cloned from *Escherichia coli*, enabling the conversion of propionyl-CoA into *n*-propanol (Ammar et al. 2013). This strategy significantly reduced byproduct accumulation through carbon source regulation, fermentation parameter optimization, and the knockout of competitive pathways (e.g., acetate synthesis pathway) (Suwannakham et al. 2006).

#### Yeast

Yeast can synthesize n-propanol under natural fermentation conditions (Zironi et al. 1993). Under pure conditions, *Kloeckera apiculata* and *Hanseniaspora guilliermondii* produced low concentrations of n-propanol (14 mg/L and 17 mg/L, respectively), whereas *Saccharomyces cerevisiae* reached a significantly higher concentration of 59 mg/L. In co-culture conditions (9:1 ratio of *H. guilliermondii* or *K. apiculata* with *S. cerevisiae*), the n-propanol concentration significantly increased to 81 mg/L and 82 mg/L, respectively (Zironi et al. 1993). During wine fermentation, both *S. cerevisiae* and *S. bayanus* produced n-propanol, with *S. cerevisiae* reaching a concentration of 182 mg/L, significantly higher than other strains (Antonelli et al. 1999). The key precursor for n-propanol biosynthesis in Yeast is 2-ketobutyrate, and its metabolic pathway directly influences n-propanol yield (Atsumi and Liao 2008; Baichwal et al. 1983).

**Fig. 3:**
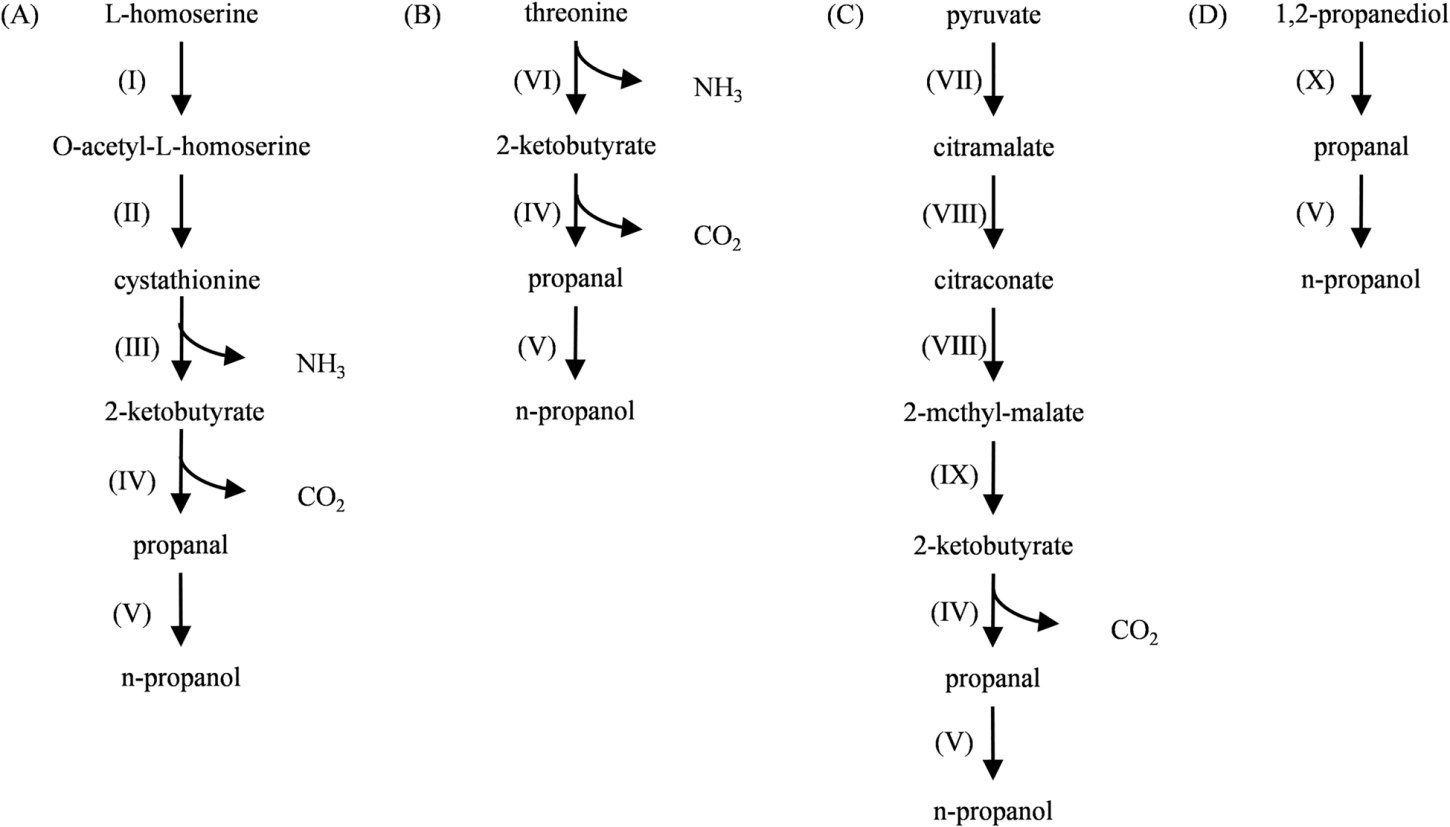
Natural propanol synthesis pathways in Yeast. **A** L-homoserine pathway; **B** threoninepathway; **C** citramalate pathway; **D** 1,2-propanediol pathway. (Ⅰ) Homoserine acetyltransferase;(Ⅱ) Putative cystathionine γ synthase; (Ⅲ) Cystathionine γ-lyase; (Ⅳ) Indolepyruvate decarboxylase; (Ⅴ) Alcohol dehydrogenase; (Ⅵ) Threonine deaminase (or threonine dehydratases); (Ⅶ) Citramalate synthase; (Ⅷ) 3-Isopropylmalate hydro-lyase; (Ⅸ) 3-Isopropylmalate dehydrogenase; (Ⅹ) Propanediol dehydratase

Currently, n-propanol can be synthesized in Yeast via three distinct metabolic pathways, depending on the source of 2-ketobutyrate: the L-homoserine pathway (Fig. 3A), the threonine pathway (Fig. 3B), and the citramalate pathway (Fig. 3C). In the L-homoserine pathway, L-homoserine is converted into O-acetyl-L-homoserine, which subsequently forms cystathionine and is further metabolized into 2-ketobutyrate. The threonine pathway involves threonine transaminase or threonine dehydratase, which catalyzes the conversion of threonine into 2-ketobutyrate (Celińska et al. 2018; Hazelwood et al. 2008). The citramalate pathway (the pyruvate pathway) is the most extensively studied n-propanol biosynthesis route in *S.cerevisiae* (El-Dalatony et al. 2019). In this pathway, pyruvate is converted into citramalate, which is then further metabolized into citraconate. Citraconate transforms into 2-methylmalate via isopropylmalate synthase and is ultimately converted into 2-ketobutyrate. Within Yeast, 2-ketobutyrate produced through these pathways is decarboxylated by indolepyruvate decarboxylase to generate propanal and CO_2_. Propanal is subsequently reduced to n-propanol by alcohol dehydrogenase (Wang et al. 2021b). Therefore, increasing endogenous 2-keto acid decarboxylase activity can promote the conversion of 2-ketobutyrate into n-propanol (Nishimura et al. 2018). Overexpressing threonine dehydratase to enhance threonine biosynthesis, promoting the conversion of aspartate to threonine, or eliminating competing pathways that divert threonine metabolism toward glycine synthesis, can significantly boost n-propanol production. Additionally, n-propanol can also be generated via the 1,2-propanediol pathway (Fig. 3D) (Wang et al. 2025).

During ethanol synthesis, for every molecule of glucose consumed, Yeast obtains two molecules of ATP through glycolysis and maintains the balance of NAD^+^/NADH by producing ethanol, enabling this process to cycle rapidly. In contrast, Yeast synthesizes propanol without generating additional ATP. It even requires energy consumption (for example, during the transamination step). Therefore, this is a branch metabolic pathway for Yeast when amino acids are in excess, and the yield of propanol is very low.

#### Lactobacillus

*Lactobacillus* is a group of Gram-positive, non-spore-producing, and rod-shaped bacteria widely found in fermented foods, animal intestines, and plant surfaces. Current research on *Lactobacillus* primarily focuses on lactic acid production. However, the potential of *Lactobacillus* to synthesize n-propanol under specific conditions, as demonstrated by (Keenan and Lindsay 1967), is an exciting prospect for future applications. Their investigation into the dehydrogenase activity of *Lactobacillus* revealed that *L. brevis* produced n-propanol as a metabolic product when grown in milk cultures.

In addition to synthesizing n-propanol via the threonine pathway, Lactobacillus can also utilize 1,2-propanediol produced by glycolytic metabolism to generate propanal under the action of propanediol dehydratase. Propanal is then reduced to n-propanol by 1,3-propanediol dehydrogenase or 1-propanol dehydrogenase (Matsubara et al. 2016; Siebert and Wendisch 2015). During the production of Chinese Baijiu, *Lactobacillus*, *Saccharomyces*, *Clostridium*, and other microorganisms jointly participate in synthesizing n-propanol. *L. pentosus* and *L. vaginalis* produced n-propanol 4.3–5.2 mg/L, close to the ability of *S. cerevisiae* and *Pichia fermentans* (Lu et al. 2019; Siebert and Wendisch 2015). Although the traditional view holds that *S. cerevisiae* is the primary producer of n-propanol (Tian et al. 2020; Wu et al. 2016), recent metagenomic and metabolomic analyses identified *L. panis*, *Pediococcus pentosaceus*, and *Bacillus alkalitelluris* as key contributors to n-propanol formation via 1,2-propanediol dehydrogenase activity, with *L. panis* being a core functional microorganism in this process (Wang et al. 2025). Beyond alcoholic fermentation, *Lactobacillus*, for example, *L. buchneri*, enhances n-propanol production to 7.16 g/kg dry matter in silage and beer brewing (Kristensen et al. 2010). Although *Lactobacillus* plays an essential role in n-propanol biosynthesis, its metabolic mechanisms are complex and significantly influenced by environmental factors, which remain unclear (Zhang et al. 2025).

### Genetically engineered strains

Natural fermentation-based biopropanol production typically yields low concentrations, making it insufficient for industrial-scale applications. Researchers have increasingly employed genetic and metabolic engineering strategies to address this limitation, optimizing propanol biosynthesis pathways and enhancing production yields. The most commonly engineered microbial hosts for propanol production, including *Clostridium*, *E. coli*, and *S. cerevisiae*, have been chosen for their well-characterized genetic backgrounds, ease of genetic manipulation, and metabolic versatility. These microorganisms are ideal hosts, playing a crucial role in the research. By introducing exogenous genes, optimizing metabolic networks, regulating key enzyme expression, and eliminating competing pathways (Table 2), researchers have significantly improved propanol yield and productivity.

**Table 2:** Biopropanol production by different genetically engineered strains

Strains	Cultivation conditions	Concentration (mmol/L)	Yield (g/g)	Productivity [g/(L h)]	References
*C. acetobutylicum* ATCC 824	Based on IBE fermentation Introduction of *adc*, *ctfA* and *ctfB* to enhance acetone synthesis Introduction of *adhB-593* from *C. beijerinckii* NRRL B593 to convert acetone to isopropanol Batch culture	146.4	–	0.80	(Collas et al. 2012)
*C. acetobutylicum* ATCC 824	Based on IBE fermentation Introduction of *adhB-593* from *C. beijerinckii* NRRL B593 Construction of synthetic acetone operon containing *adc*, *ctfA* and *ctfB* genes Batch culture	73.2	0.1	–	(Joungmin et al. 2012)
*C. acetobutylicum* ATCC 824	Based on IBE fermentation Introduction of *adhB-593* from *C. beijerinckii* NRRL B593 Construction of synthetic acetone operon containing *adc*, *ctfA* and *ctfB* genes The solvent is removed by gas stripping Fed-batch culture	174.7	0.09	0.79	(Joungmin et al. 2012)
*C. acetobutylicum* BKM19	Based on IBE fermentation Introduction of *sadh* and *hydG* from *C. beijerinckii* NRRL B593.The expression of *sadh* and *hydG* genes is controlled using a thioylase promoter (*Pthl*) to ensure that expression begins in the acidogenic phase in preparation for subsequent acetone reduction Batch culture	59.9	–	–	(Jang et al. 2013)
*C. acetobutylicum* ATCC 824	Based on IBE fermentation Construction of synthetic isopropanol operon containing *sadh*, *adc*, *ctfA* and *ctfB* genes Synthetic isopropanol operon was introduced into butyrate kinase deletion strain Batch culture	78.2	0.11	0.8	(Dusséaux et al. 2013)
*C. acetobutylicum* DSM 792	Based on IBE fermentation Introduction of *adh* from *C. beijerinckii* NRRL B593 Construction of synthetic acetone operon containing *adc*, *ctfA* and *ctfB* genes Use of the allele-coupled exchange approach to integrate *adh* genes into chromosomes of *C. acetobutylicum* DSM 792 Shake flask culture	41.8	–	–	(Bankar et al. 2015)
*C. acetobutylicum* XY16	Based on IBE fermentation Introduction of *sadh* from *C. beijerinckii* NRRL B593 Batch culture	100.2	–	–	(Wang et al. 2018a)
*C. acetobutylicum* ATCC 824	Based on IBE fermentation The *pSOL1* large plasmid (carrying the solvent synthesis-related gene) was integrated into the *pyrE* site of the chromosome to achieve stable integration through four cross-recombination events The *sadh* and *hydG* genes were inserted into the integrated *pSOL1* and their expression was controlled by the natural *thlA* promoter Continuous fermentation	256.2	0.31	–	(Ehsaan et al. 2024)
*S. elongatus* PCC 7942	Based on IBE fermentation Introduction of *thl* and *adc* from *C. acetobutylicum* ATCC 824, *atoAD* (acetoacetyl-CoA transferase) from *E. coli* K-12 MG1655 and *adh* from *C. beijerinckii* NRRL B593 Under nitrogen and phosphorus restriction conditions Batch culture	0.4	–	0.003	(Kusakabe et al. 2013)
*S. elongatus* PCC 7942	Based on IBE fermentation Introduction of *thl*, *adc*, *atoAD* and *sadh* Two phase fermentation Batch culture	2.4	–	–	(Hirokawa et al. 2015)
*S. elongatus* PCC 7942	Based on IBE fermentation Introduction of *thl*, *adc*, *atoAD* and *sadh* Introduction of *pta* from *E. coli* to optimize the expression system Shake flask culture	0.6	–	–	(Hirokawa et al. 2017)
*C. necator Re2133*	Based on IBE fermentation Introduction of *adc* and *adh* from *Clostridium* Overexpression of *thl* and *ctf* Shake flask culture	57.2	0.24	0.044	(Grousseau et al. 2014)
*C. necator Re2133*	Based on IBE fermentation Introduction of *thl*, *adc*, *ctfAB* and *adh* Overexpression of *GroESL* to improve the activity of *adc* and *adh* Fed-batch culture	151.4	–	0.047	(Marc et al. 2017)
*Acetobacterium woodii*	Based on IBE fermentation Introduction of genes from *C. acetobutylicum* and *C. aceticum* Supplyment of continuous gas Continuous agitation tank culture	14.5	–	–	(Arslan et al. 2022)
*Corynebacterium glutamicum*	Based on IBE fermentation Introduction of *thlA*, *atoAD*, *adc* and *adh* Deletion of *lah* and *ppc* to reduce lactic acid and succinic acid Fed-batch culture	170.5	0.34	0.1	(Ko et al. 2022a)
*Moorella thermoacetica*	Based on IBE fermentation Introduction of *sadh* from *Thermoanaerobacter pseudoethanolicus* Deletion of *pduL2* to reduce acetic acid Batch culture	8.1	0.81	0.076	(Kato et al. 2024)
*E. coli* TA11	Based on IBE fermentation Overexpression of *thl*, *ctfAB* and *adc* from *C. acetobutylicum*, and *adhB-593* from *C. beijerinckii* NRRL B593 Shake flask culture	81.6	0.435	0.41	(Hanai et al. 2007)
*E. coli* JM109	Based on IBE fermentation Overexpression of *thl*, *ctfAB* and *adc* from *C. acetobutylicum* ATCC 824, and *adhB-593* from *C. beijerinckii* NRRL B593 Aerobic fed-batch culture	227.0	0.51	0.619	(Jojima et al. 2008)
*E. coli* TA76	Based on IBE fermentation Overexpression of *thl* and *adc* from *C. acetobutylicum* ATCC 824, endogenous *atoAD* from *E. coli* K-12 MG1655, and *adhB-593* from *C. beijerinckii* NRRL B593 Gas stripping to avoid growth inhibition by isopropanol Fed-batch culture	143.0	0.674	0.61	(Inokuma et al. 2010)
*E. coli* TA212	Based on IBE fermentation Introducing *Tfu-Blc* fused protein expression system and the synthetic pathway for isopropanol production Fermentation material is cellobiose Batch culture	69.0	0.248	–	(Soma et al. 2012)
*E. coli* TA1415	Based on IBE fermentation Switching *gltA* off to redirect carbon flux from the TCA cycle to the anabolic pathway of isopropyl alcohol Shake flask culture	49.9	0.48	0.04	(Soma et al. 2014)
*E. coli* MG1655	Based on IBE fermentation Overexpression of *thlA* and *adc* from *C. acetobutylicum*, *atoAD* from *E. coli*, and *adh* from *C. beijerinckii* Enhancement of the acetate assimilation pathway coupled with cofactor engineering strategy Biologically produced acetate from syngas were used as carbon source Shake flask culture	24.5	0.56	–	(Yang et al. 2020b)
*E. coli* MG1655	Based on IBE fermentation Acetoacetyl-CoA is condensed with acetyl-CoA to generate 3-hydroxy-3-methylglutaryl-CoA (HMG-CoA) by HMG-CoA synthase, and then catalyzed by HMG-CoA lyase to generate acetoacetate Blocking the degradation pathway of acetoacetyl-CoA Batch culture	16.0	–	–	(Zhou et al. 2022)
*Candida utilis* TMS272	Based on IBE fermentation Introduction of *ctfAB* from *C. acetobutylicum*, *adc* and *sadh* from *C. beijerinckii* Overexpression of *ACS1*or *ACS2* (Acetyl-CoA synthetase) and *ERG10* (Acetoacetyl-CoA thiolase) Deletion of *PDC1* (pyruvate decarboxylase) Fed-batch culture	453.0	0.415	0.13	(Tamakawa et al. 2013)
*Yarrowia lipolytica* Po1g	Based on IBE fermentation Introduction of *nphT7* (Acetoacetyl-CoA synthase), *atoAD*, *adc*, *adh* Fermentation material is 73.53 g/L crude glycerol Shake flask culture	8.3	–	–	(Shi et al. 2022)
*Yarrowia lipolytica* Po1g	Based on IBE fermentation Introduction of *nphT7*, *atoAD*, *adc*, *adh* Fermentation material is 73.53 g/L crude glycerol Fed-batch culture	26.6	–	–	(Shi et al. 2022)
*E. coli* BW25113	Based on the threonine pathway Overexpression of *kivd* from *L. lactis*, *ADH2* from *S. cerevisiae* and *ilvA*, *thrA* *BC* from *E. coli* Knocking out competing genes (*metA*, *tdh*, *ilvB*, *ilvI*) related to threonine metabolism Shake flask culture	16.6	0.11	0.01	(Shen and Liao 2008)
*E. coli* DE3	Based on the citramalate pathway Overpression of *CimA* from *Methanococcus jannaschii* Shake flask culture	58.2	0.54	0.03	(Atsumi and Liao 2008)
*E. coli* BW25113	Based on 1,2-propanediol pathway Overexpression of *MgsA* from *Bacillus subtilis*, *YdjG* from *E. coli*, *budC* from *Klebsiella pneumoniae* and *ppdABC* from *Klebsiella oxytoca* Shake flask culture	4.2	–	0.005	(Jain and Yan 2011)
*E. coli* W3110	Based on the synergistic effect of the threonine pathway and the citramalate pathway Introduced resistant *ilvA* gene encoding threonine dehydratase and Deleted the competing metabolic pathway genes Overexpression of *CimA*, *ackA* (acetate kinase A/propionate kinase II) and *adhE* Deletion of *rpoS* (stationary phase sigma factor) Aerobic fed-batch culture	179.7	0.32	0.144	(Jun Choi et al. 2012)
*E. coli* BW25141	Based on the succinyl-CoA pathway Overexpression of *sbm*, *ygfD*, *ygfG*, *sucCD*, *adhE* from *C. acetobutylicum* Supplement of succinate Shake flask culture	2.5	–	–	(Srirangan et al. 2013)
*E. coli* BW25113	Based on the synergistic effect of the threonine pathway and citric acid pathway Overexpression of key genes of the threonine pathway and citric acid pathway Deletion of genes related to threonine metabolism (such as *thrB*) Deletion of genes in acetic acid synthesis pathway Introduction of NADPH-dependent enzymes in the citric acid pathway Shake flask culture	133.1	0.45	0.12	(Shen and Liao 2013)
*E. coli* BW25113	Based on the succinyl-CoA pathway Activation of the *Sbm* operon (*sbm*, *ygfG*, *ygfH*, *ygfD*) Deletion of *ldhA* (lactate dehydrogenase) and *pykF* (pyruvate kinase I/II) Carbon source is glycerol Anaerobic fed-batch culture	112.5	–	–	(Srirangan et al. 2014)
*E. coli* BW25113	Based on 1,2-propanediol pathway Introduction of *ppdABC*, *adhE*, *fdhl* Deletion of *zwf*、*tpiA*、*ldhA*、*gloA*、*adhE* Shake flask culture	48.4	–	–	(Jain et al. 2015)
*E. coli* BW38029	Based on 1,2-propanediol pathway Overexpression of *pduCDE*, *pduQ*, p*duGHOQS*, *pAC_sbdhaK* Carbon source is glucose Shake flask culture	16.1	0.36	–	(Matsubara et al. 2016)
*E. coli* BW38029	Based on 1,2-propanediol pathway Overexpression of *pduCDE*, *pduQ*, p*duGHOQS*, *pAC_sbdhaK* Carbon source is rhamnose Shake flask culture	29.9	0.81	–	(Matsubara et al. 2016)
*E. coli* BW38029	Based on 1,2-propanediol pathway Overexpression of *pduCDE*, *pduQ*, p*duGHOQS*, *pAC_sbdhaK* Carbon source is glycerol Shake flask culture	12.6	0.27	–	(Matsubara et al. 2016)
*E. coli* MG1655	Based on the threonine pathway Overexpression of *kivd* from *L. lactis*, *ADH2* from *S. cerevisiae* and *ilvA*, l*euABCD*, *thrABC* from *E. coli* Deletion of *tdh* Fed-batch culture	22.5	–	–	(Liu et al. 2019b)
*E. coli* MG1655	Based on the succinyl-CoA pathway Introduction of key genes from *P. acidipropionici* Deletion of *ldhA*, *adhE*, *pflB* Shake flask culture	4.5	–	–	(Gonzalez-Garcia et al. 2020)
*E. coli* DH5α	Based on the threonine pathway Tunable expression systems are used to optimize genetic circuits Shake flask culture	1.6	–	–	(Yoo et al. 2020)
*E. coli* BuT-3E	Introduction of *atoAD* and *adhE2* Enhancement of *aceEF* operon (pyruvate dehydrogenase complex) and *acs* gene (acetyl-CoA synthetase) Deletion of *ldhA* Shake flask culture	89.9	–	0.15	(Lin et al. 2021b)
*E. coli* SYN12	Based on the threonine pathway and the citramalate pathway Overexpression of *kivD*, *YqhD* and *tpiA* Deletion of *avtA* Fed-batch culture	84.9	0.142	–	(Ohtake et al. 2022)
*S. cerevisiae* YPH499	Based on the synergistic effect of the threonine pathway and the citramalate pathway Overexpression of *tdcB* (threonine dehydratase) and *thrABC* Introduction of *CimA* from *Methanocaldococcus jannaschii*, *leuC* from *Clostridium beijerinckii* and *leuD* from *E. coli* Deletion of *GLY1* (threonine aldolase) Shake flask culture	1.6	0.005	0.0014	(Nishimura et al. 2018)
*S. cerevisiae* YPH499	Based on the synergistic effect of the threonine pathway and the citramalate pathway Overexpression of *tdcB* (threonine dehydratase) and *thrABC* Introduction of *CimA* from *Methanocaldococcus jannaschii*, *leuC* from *C. beijerinckii* and *leuD* from *E. coli* Deletion of *GLY1* (threonine aldolase) Shake flask culture	3.0	0.009	0.0019	(Nishimura et al. 2018)

#### Isopropanol synthesis using genetically modified strains

Synthesis of isopropanol in microorganisms occurs primarily in the acetone-dependent isopropanol pathway in *Clostridium*. This pathway involves the condensation of acetyl-CoA to form acetoacetyl-CoA, which is subsequently converted to acetoacetate. This compound is then decarboxylated to form acetone. Finally, acetone is reduced to isopropanol by primary/secondary alcohol dehydrogenase. However, isopropanol production in natural *Clostridium* is low, usually not exceeding 25% of total alcohols (George et al. 1983). As a result, researchers have begun to modify microorganisms using genetic engineering.

**Fig. 4:**
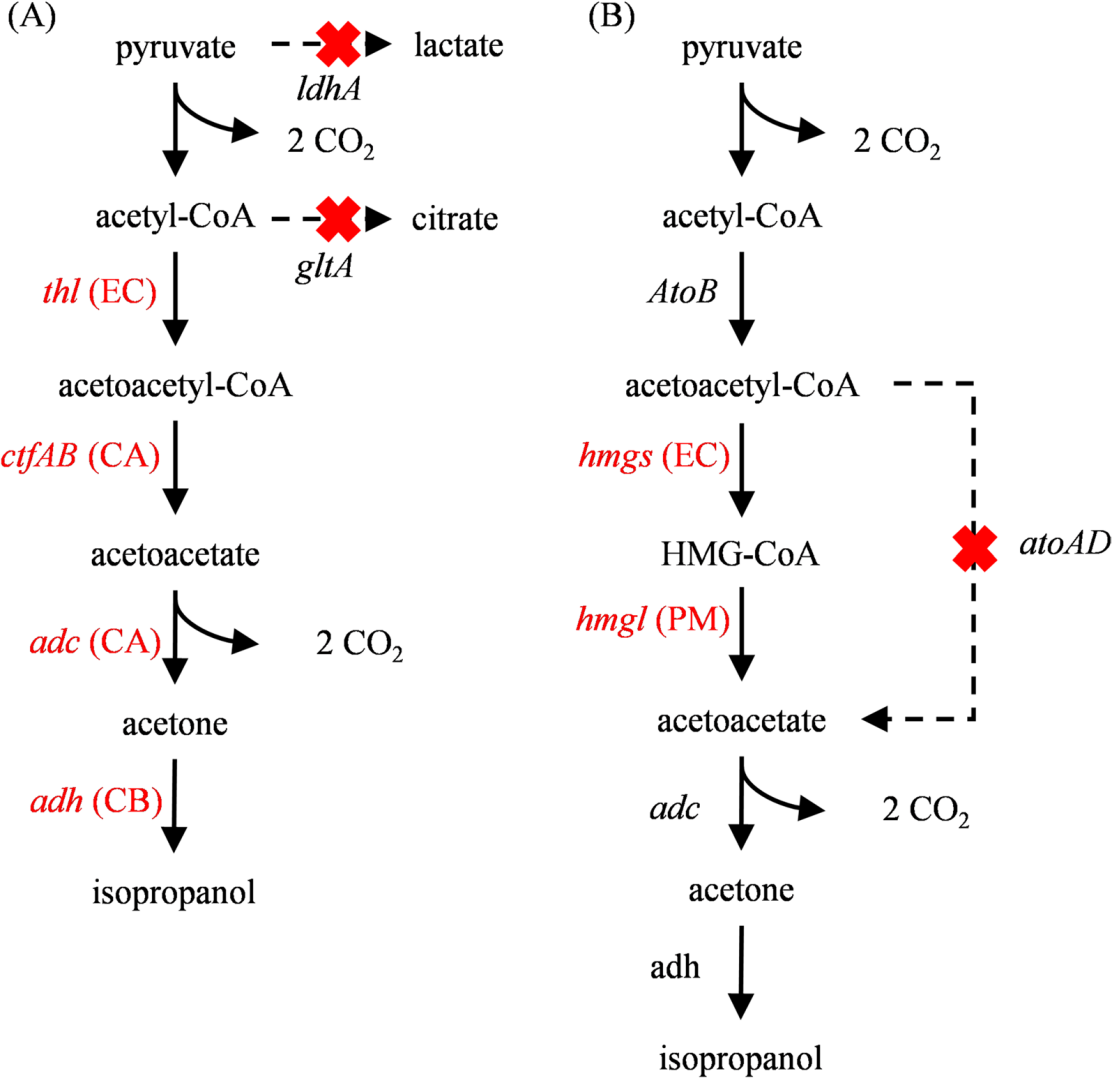
Isopropanol synthesis pathway in genetic engineering. **A** Conventional synthesis route **B** Novel synthesis route. Genes that are overexpressed or exogenously introduced are shown in red font, whereas genes not subject to overexpression are shown in black. Cross mark indicates that the pathway was blocked via gene deletion. EC, *E. coli*; CA, *C. acetobutylicum*; CB, *C. beijerinckii*; PM, *Pseudomonas mevalonii*

Due to its well-characterized genetic background, rapid growth, and ease of genetic manipulation, *E. coli* has become a key host for metabolic engineering. To enable acetone biosynthesis (a precursor to isopropanol) in *E. coli*, a synthetic acetone operon, ace4, was constructed, incorporating key genes from *C. acetobutylicum* ATCC 824, including acetoacetate decarboxylase (*adc*), CoA-transferase (*ctfAB*), and acetoacetyl-CoA thiolase (*thl*) (Bermejo et al. 1998). These genes were cloned into the plasmid *pACT* and expressed in three different *E. coli* strains, resulting in acetone production of up to 93 mM, comparable to that of *C. acetobutylicum* ATCC 824. Building on this, researchers further introduced an acetone reduction pathway to achieve isopropanol synthesis. A primary/secondary alcohol dehydrogenase (*adhB-593*) from *C. beijerinckii* NRRL B593 was expressed in *E. coli*, resulting in an isopropanol production titer of 5 g/L (Hanai et al. 2007). By optimizing the metabolic pathway and overexpressing acetoacetyl-CoA thiolase (*thl*), replacing acetoacetyl-CoA:acetate CoA-transferase (*atoB*) in the native *Clostridium* pathway (Fig. 4A), isopropanol production increased significantly. The engineered *E. coli* strain produced 81.6 mmol/L isopropanol, with a production efficiency of 43.5% (mol/mol), outperforming known natural producers. This system was further enhanced to achieve isopropanol production up to 227 mmol/L under aerobic fed-batch fermentation (Jojima et al. 2008). Additionally, engineered *E. coli* strains have demonstrated the ability to utilize alternative carbon sources such as fatty acid-rich substrates (Dellomonaco et al. 2010), cellobiose (Soma et al. 2012), and acetate (Yang et al. 2020b), showcasing their metabolic flexibility. Knocking out competing pathways can also increase the yield. A metabolic toggle switch was developed in *E. coli* by knocking out *gltA*, which encodes citrate synthase, thereby redistributing the TCA cycle flux toward isopropanol biosynthesis and increasing the yield and production by 3.7-fold and 3.1-fold, respectively (Soma et al. 2014). Similarly, a tunable cell density sensor was employed to autonomously redirect metabolic flux, enhancing isopropanol production (Soma and Hanai 2015).

To further enhance yield, a novel isopropanol biosynthesis pathway was constructed, in which acetoacetyl-CoA and acetyl-CoA are condensed by HMG-CoA synthase to generate 3-hydroxy-3-methylglutaryl-CoA, which is subsequently cleaved by HMG-CoA lyase to produce acetoacetate (Fig. 4B) (Zhou et al. 2022). Using this pathway, the engineered *E. coli* strain achieved a sevenfold increase in isopropanol production from glycerol. By knocking out *pta-ackA* (blocking acetyl-CoA conversion to acetate) and *atoAD* (inhibiting acetoacetate degradation), isopropanol production increased to 964 mg/L, a 7.1-fold improvement over the control strains, demonstrating the efficiency of this novel pathway.

*C. acetobutylicum* naturally produces acetone, butanol, and ethanol. Since acetone is not a viable biofuel, researchers applied metabolic engineering to convert acetone into isopropanol, resulting in an isopropanol-butanol-ethanol (IBE) mixture suitable for biofuel applications (Collas et al. 2012; Jang et al. 2013; Joungmin et al. 2012). Acetone was successfully converted into isopropanol by introducing *adh* from *C. beijerinckii* NRRL B593. Additionally, researchers introduced *adc* and *ctfAB* to enhance the acetone biosynthesis pathway. Under uncontrolled pH conditions, the engineered *C. acetobutylicum* ATCC 824 produced 24.4 g/L total solvents (IBE), with isopropanol reaching 8.8 g/L (Collas et  al. 2012). In a fed-batch fermentation combined with gas stripping, the strain produced 35.6 g/L of IBE, with isopropanol reaching 10.5 g/L (Joungmin et al. 2012). Further improvements have been achieved using integrated DNA technology, with nearly 50% of acetone converted into isopropanol, reaching a concentration of 4.98 g/L by controlling pH and supplementing calcium carbonate to optimize intracellular NAD(P)H levels (Bankar et al. 2015). However, due to *Clostridium*'s higher tolerance to butanol, research has primarily focused on biobutanol production rather than isopropanol. The potential of *Clostridium* as an industrial isopropanol producer requires further exploration.

Yeast species such as *Saccharomyces cerevisiae* and *Candida utilis* have been explored for isopropanol biosynthesis due to their robust metabolism and tolerance to complex fermentation conditions. The acetone-dependent isopropanol biosynthesis pathway was reconstructed in *C. utilis* by incorporating *ctfAB*, *adc*, and *sadh* genes from *Clostridium acetobutylicum*, thereby establishing an acetone-to-isopropanol conversion module (Tamakawa et al. 2013). Overexpressing acetyl-CoA synthase (*ACS1* or *ACS2*) and acetoacetyl-CoA thiolase (*ERG10*) and knocking out pyruvate decarboxylase (*PDC1*) significantly increased isopropanol yield. Under fed-batch fermentation with 50 g/L glucose and 40 g/L calcium carbonate, the engineered strain achieved an isopropanol titer of 27.2 g/L, with a molar yield of 41.5% and a productivity of 0.13 g/(L h). Although Yeast still lags behind *E. coli* in efficiency, its ability to utilize complex substrates offers cost advantages. For example, *Yarrowia lipolytica* was engineered to produce isopropanol using crude glycerol as a low-cost carbon source, achieving a yield of 1.60 g/L, which is comparable to that from pure glycerol fermentation (1.94 g/L) (Shi et al. 2022).

Besides the above-mentioned hosts, several other microbes have been engineered for isopropanol production. An isopropanol biosynthesis pathway was constructed in *Synechococcus elongatus* PCC 7942 by introducing the *thl*, *atoAD*, *adc*, and *adh* genes, resulting in 26.5 mg/L isopropanol under nitrogen- and phosphorus-limiting conditions; this yield was subsequently optimized to 146 mg/L (Kusakabe et al. 2013). *Cupriavidus necator*, a β-proteobacterium capable of utilizing diverse carbon sources, was engineered to produce 1.2 g/L isopropanol (Grousseau et al. 2014). Isopropanol production by *Acetobacterium woodii* under gas fermentation reached 14.5 mmol/L (Arslan et al. 2022). Additionally, *Corynebacterium glutamicum* was metabolically optimized to enhance isopropanol synthesis, achieving a high titer of 10.25 g/L (Ko et al. 2022a).

#### N-propanol synthesis using genetically modified strains

Genetic engineering has enabled the development of multiple metabolic pathways for n-propanol biosynthesis, primarily using *E. coli* as the host.

**Fig. 5:**
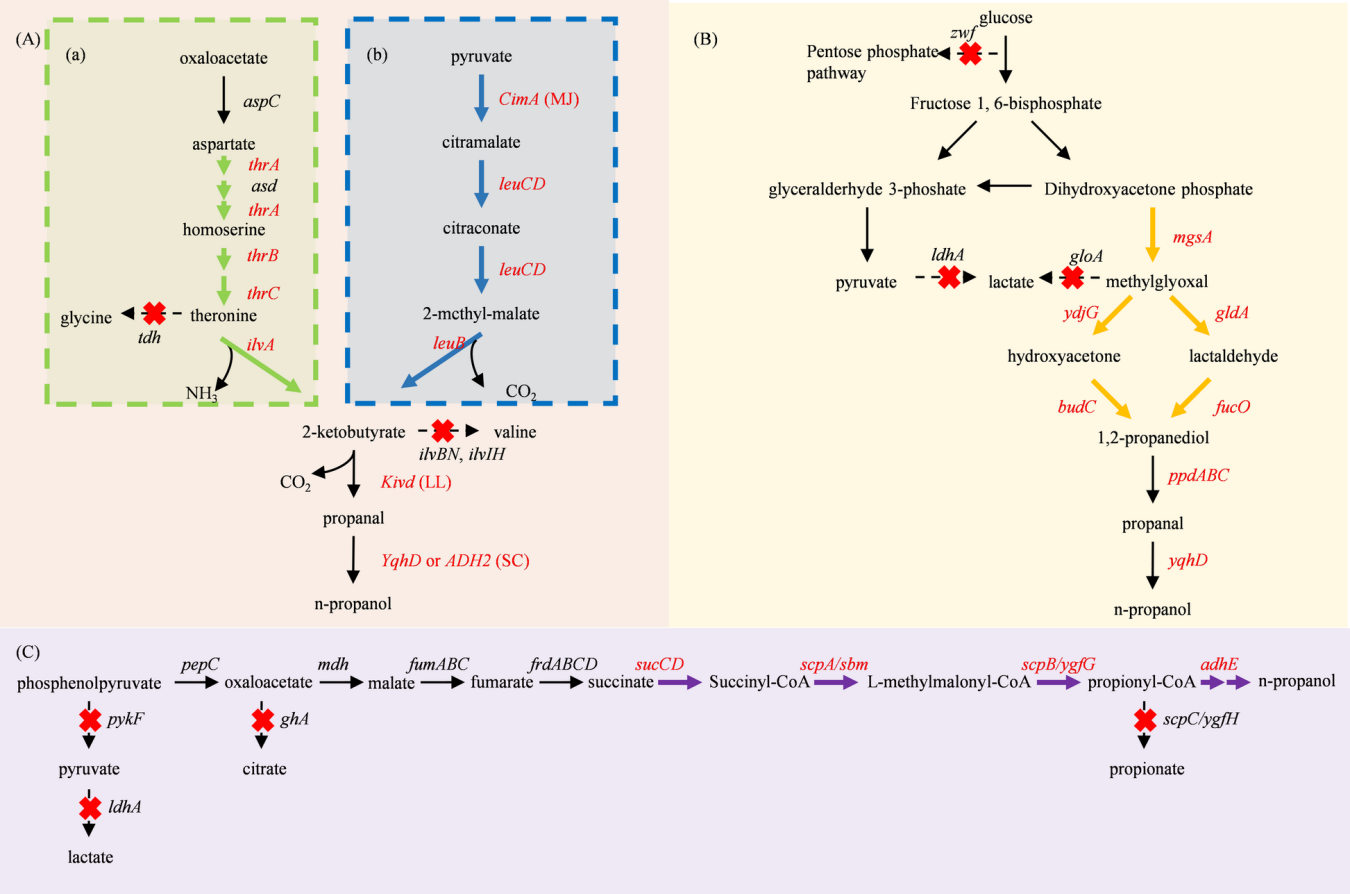
N-propanol synthesis pathway in genetic engineering. **A** 2-Ketobutyrate pathway, (**a**) threonine pathway, (**b**) citramalate pathway; **B** 1,2-propanediol pathway; **C** succinyl-CoA pathway. Genes that are overexpressed or exogenously introduced are shown in red font, whereas genes not subject to overexpression are shown in black. Cross mark indicates that the pathway was blocked via gene deletion. LL, *L. lactis*; SC, *S. cerevisiae*; MJ, *Methanococcus jannaschii*

N-propanol synthesis in *E. coli* via the threonine pathway (Fig. 5A) was first demonstrated by (Shen and Liao 2008). The key to this pathway is the removal of feedback inhibition in threonine biosynthesis, thereby increasing the supply of 2-ketobutyrate. Researchers overexpressed key genes in the threonine biosynthesis pathway (*ilvA*, *thrABC*), significantly enhancing the conversion of threonine to 2-ketobutyrate. Subsequently, they introduced 2-keto acid decarboxylase (*Kivd*) from *Lactococcus lactis* and secondary alcohol dehydrogenase (*ADH2*) from *S. cerevisiae*, successfully converting 2-ketobutyrate into n-propanol, with an initial yield of approximately 60 mg/L. To further improve production, competitive genes related to threonine metabolism (*metA*, *tdh*, *ilvB*, *ilvI*, etc.) were knocked out to minimize 2-ketobutyrate diversion, ultimately increasing n-propanol yield to 1 g/L. In most microorganisms, 2-ketobutyrate is primarily synthesized through the threonine pathway. Additionally, by implementing a protein scaffold strategy, carbon flux was redirected toward the n-propanol synthesis pathway. Under conditions of pH 7 and 37 °C, the recombinant strain produced 3.8 g/L of 1-propanol via the protein scaffold without the need for further metabolic engineering. Optimization of the enzyme ratios (*ilvA*, *Kivd*, and *YqhD* at 4:5:1) and the introduction of a scaffold containing GDB, SH3, and PDZ domains further increased the yield to 4.5 g/L of n-propanol (Tran et al. 2025).

However, some organisms possess an alternative route known as the citramalate pathway (Fig. 5A) (Howell et al. 1999; Westfall et al. 1983). The *CimA* gene from *Methanococcus jannaschii* was introduced into *E. coli* to bypass threonine biosynthesis, which significantly improved n-propanol production efficiency from glucose. (Atsumi and Liao 2008).

The 1,2-propanediol pathway (Fig. 5B) was expanded by incorporating additional enzymatic steps, which enabled the synthesis of n-propanol (Jain and Yan 2011). This process involves: (1) Conversion of dihydroxyacetone phosphate to methylglyoxal using methylglyoxal synthase (*MgsA*) from *Bacillus subtilis*. (2) Reduction of methylglyoxal to hydroxyacetone by endogenous methylglyoxal reductase (*YdjG*) in *E. coli*. (3) Further reduction of hydroxyacetone to 1,2-propanediol using secondary alcohol dehydrogenase (*BudC*) from *Klebsiella pneumoniae*. (4) Dehydration of 1,2-propanediol to propanal by 1,2-propanediol dehydratase (*ppdABC*) from *Klebsiella oxytoca*. (5) Reduction of propanal to n-propanol by endogenous alcohol dehydrogenase in *E. coli*. The engineered strain achieved an initial n-propanol yield of 0.25 g/L after 48 h of fermentation. Further improvements, achieved through fusion protein construction, NADH regeneration optimization, adaptive strategies, and dual-strain systems, increased the yield to 2.91 g/L (Jain et al. 2015).

An n-propanol synthesis strategy based on the succinyl-CoA pathway (Fig. 5C) was developed by (Srirangan et al. 2013). The pathway expresses the *sbm* operon (*sbm*, *ygfD*, *ygfG*) in *E. coli* to convert succinyl-CoA into propionyl-CoA. A bifunctional aldehyde/alcohol dehydrogenase (*adhE*) from *C. acetobutylicum* reduces propionyl-CoA to n-propanol. Overexpressing succinyl-CoA synthetase (*sucCD*) increased succinyl-CoA supply, boosting n-propanol production from 47 mg/L to 103 mg/L. Supplementation with succinate further increased the yield to 168 mg/L. In a glycerol-fed batch fermentation system, knocking out lactate dehydrogenase (*ldhA*) and pyruvate kinase (*pykF*) optimized carbon flux, converting over 70% of glycerol into solvents (ethanol and n-propanol), achieving an n-propanol yield of 6.76 g/L (Srirangan et al. 2014).

A hybrid approach utilizing the threonine and citramalate pathways has also been developed for the efficient production of n-propanol. (Jun Choi et al. 2012) found that using a threonine-overproducing *E. coli* strain as a base, systematic metabolic modifications (including the introduction of a feedback-resistant *ilvA* gene encoding threonine dehydratase to eliminate isoleucine-mediated inhibition, and the knockout of competing genes (*ilvIH, ilvBN*) to reduce 2-ketobutyrate diversion) significantly improved n-propanol synthesis. Overexpression of *CimA* to enhance 2-ketobutyrate supply and overexpression of *ackA* to convert propionyl phosphate into propionate, subsequently leading to n-propanol synthesis. Introduction of a mutant *adhE* gene and knockout of *rpoS* to reduce acetate accumulation and enhance threonine metabolism. Under aerobic conditions, the engineered strain achieved 1.38 g/L n-propanol with a productivity of 0.077 g/(L h) in shake flask fermentation. In fed-batch fermentation with 100 g/L glucose, n-propanol production increased to 10.8 g/L, with a yield of 0.107 g/g and productivity of 0.144 g/(L h). Using 40 g/L glycerol, the yield reached 10.3 g/L (0.259 g/g) with productivity of 0.083 g/(L h). The effectiveness of this dual-pathway strategy in significantly improving n-propanol production efficiency was further validated by (Shen and Liao 2013).

### Yield limitation and product inhibition

**Fig. 6:**
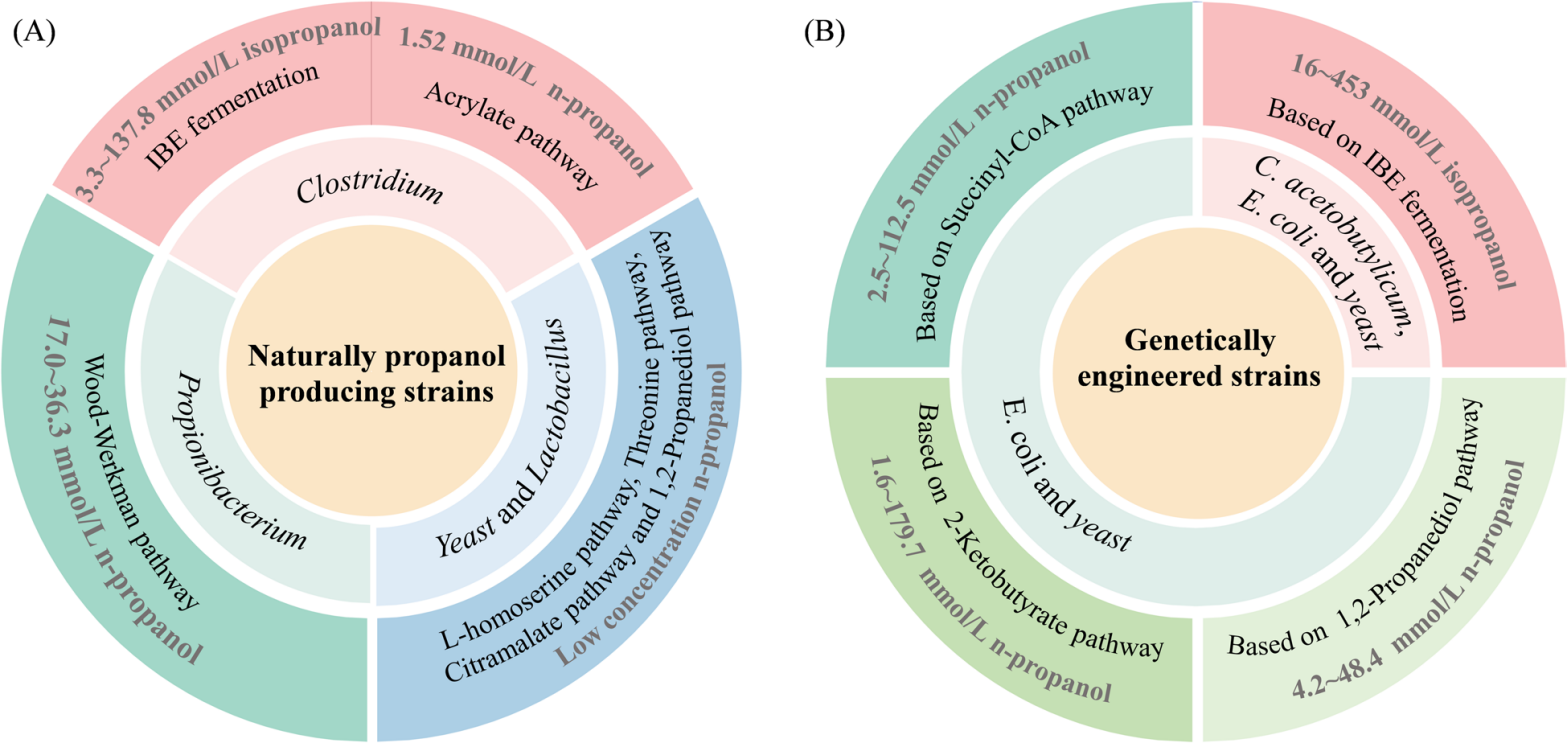
Propanol concentrations achievable via microbial fermentation. **A** Natural microorganisms; **B** genetically engineered microorganisms

In natural strains, isopropanol is primarily synthesized via the acetone-dependent metabolic pathway of certain *Clostridium* species, with representative strains such as *C.beijerinckii* and *C. acetobutylicum* capable of reaching isopropanol concentrations of up to 137.8 mmol/L (Hijosa-Valsero et al. 2019). In contrast, the ability of natural strains to produce *n*-propanol is considerably weaker (O'Quinn et al. 2025), with only trace amounts detected in *Propionibacterium*, Yeast, and *Lactobacillus*, and yields generally remaining below 40 mmol/L (Table 1), which is insufficient to meet industrial requirements. Consequently, relying solely on natural strains is inadequate for efficient and targeted *n*-propanol biosynthesis (Fig. 6A).

To enhance yield, selectivity, and substrate utilization efficiency, researchers have extensively applied metabolic engineering techniques to reprogram host organisms (such as *E. coli*, Yeast or *Clostridium*) by introducing or optimizing synthetic pathways (Fig. 6B). Through regulation of key enzymes, inhibition of competing pathways, and dynamic redistribution of metabolic flux, engineered strains not only enable stable synthesis of target products under controlled conditions but also achieve improved carbon flux distribution. Currently, isopropanol biosynthesis via genetic engineering is relatively mature, with engineered strains reaching titers of up to 27.2 g/L (Tamakawa et al. 2013). Its biosynthetic pathway is highly compatible with traditional ABE fermentation processes, offering substantial potential for industrial scaling. A close titer of 20.6 g/L on sour whey using engineered *E. coli* was reported under microaerobic conditions (Kutscha et al. 2025). In contrast, the *n*-propanol biosynthetic pathway is more complex, constrained by limitations in precursor availability, catalytic efficiency, and energy balance, and remains largely confined to the laboratory stage. Nevertheless, *n*-propanol, with its higher energy density and stronger polarity, is well-suited for high-temperature or extreme environments, presenting unique potential in biofuel applications. Despite considerable technical barriers, *n*-propanol remains a promising direction for future research.

However, both *n*-propanol and isopropanol biosynthesis still face significant challenges, including product toxicity, cellular stability, metabolic flux balance, and process scalability.

During *n*-propanol fermentation, accumulation of the target compound and intermediate metabolites, particularly propanal, markedly inhibits cell growth and metabolism. Studies indicate that when *n*-propanol concentration exceeds 25 g/L or propanal reaches 1.2 g/L, lipid peroxidation of the cell membrane occurs, resulting in a marked decline in metabolic activity (Choi Yong et al. 2014). Furthermore, toxic aldehyde intermediates disrupt membrane permeability and inhibit critical enzymatic functions, thereby limiting biosynthetic efficiency. For example, (Ohtake et al. 2022) reported that *E. coli* growth was significantly suppressed when propanal accumulation exceeded 1.2–2 g/L. To date, research on *n*-propanol tolerance mechanisms remains limited, and systematic strategies for tolerance engineering are lacking, which restricts the development of high-titer biosynthesis systems.

The inhibitory effects of isopropanol have been studied more comprehensively. In *E. coli* systems, isopropanol concentrations above 40 g/L reduce glucose uptake by 62% and decrease growth rates to 23% of the original level (Inokuma et al. 2010). Further investigations revealed that the tolerance threshold of *E. coli* MG1655 to isopropanol and its precursor acetone is approximately 300 mM. At 700 mM, most strains are severely inhibited, though some recover partial growth after prolonged cultivation (Zhou et al. 2019). In *Clostridium*, native tolerance is similarly limited, with *C. beijerinckii* growth significantly inhibited at butanol concentrations above 8 g/L (Dalal et al. 2019). Nevertheless, improvements are possible; for instance, (Gérando et al. 2016) developed an engineered strain capable of tolerating 50 g/L isopropanol via random mutagenesis and genome recombination, significantly enhancing its industrial applicability.

From a metabolic engineering perspective, the biosynthetic pathways and bottlenecks of *n*-propanol and isopropanol differ markedly. The *n*-propanol biosynthesis pathway in *E. coli* involves multiple intermediates and is prone to carbon flux diversion into non-target routes. Therefore, genes such as *tdh*, *ilvB*, *ilvI*, and *ldhA* are often deleted to eliminate competing pathways (Lin et al. 2021b; Shen and Liao 2008). The isopropanol pathway is more straightforward. It relies primarily on the two-step reduction of acetone, catalyzed by acetone decarboxylase (*adc*) and secondary alcohol dehydrogenase (*adh*) (Fig. 4A), with more concentrated carbon flux. However, this pathway is heavily dependent on the supply of NADH/NADPH, and an imbalance in reducing equivalents can impair key enzyme activities (e.g., acetone reductase), thereby limiting yields (Walther and François 2016).

In summary, microbial production of isopropanol and *n*-propanol exhibits substantial differences in pathway structure, toxicity responses, and engineering strategies. Isopropanol biosynthesis has reached a high degree of technical maturity and is supported by scalable industrial processes. n-Propanol, despite its challenges, offers greater potential due to its superior physicochemical properties. Future efforts should prioritize elucidating tolerance mechanisms, optimizing cofactor and energy metabolism, and rewiring global carbon flux to establish efficient platforms for propanol biosynthesis.

## Separation of propanol from fermentation broth

The separation of propanol represents a crucial stage in bio-propanol production, as the efficiency of this process directly influences the mitigation of product inhibition and, consequently, determines the overall economic feasibility and sustainability of the production system.

### Distillation

Distillation remains a cornerstone of chemical separation processes, accounting for approximately 90–95% of all industrial separation operations (Kong et al. 2022; Sholl and Lively 2016). In the context of industrial propanol production, distillation is commonly employed to remove impurities and improve product purity. However, its efficiency is significantly hindered by the formation of azeotropic mixtures with water, which complicates separation and limits purity. For instance, n-propanol forms an azeotrope with water at 88.5 °C containing 73.5% n-propanol (Chou et al. 1998), while isopropanol forms an azeotrope with water at 80.3–80.4 °C containing 87.4–87.7% isopropanol (Mujiburohman et al. 2006). Furthermore, industrial-grade feedstocks often involve complex ternary azeotropes, such as mixtures of water (5–45%), n-propanol (40–70%), and propyl propionate (5–25%), which require multi-column distillation and result in energy demands as high as 1,200 kWh per ton of product. To address these challenges, several advanced distillation methods have been developed.

**Fig. 7:**
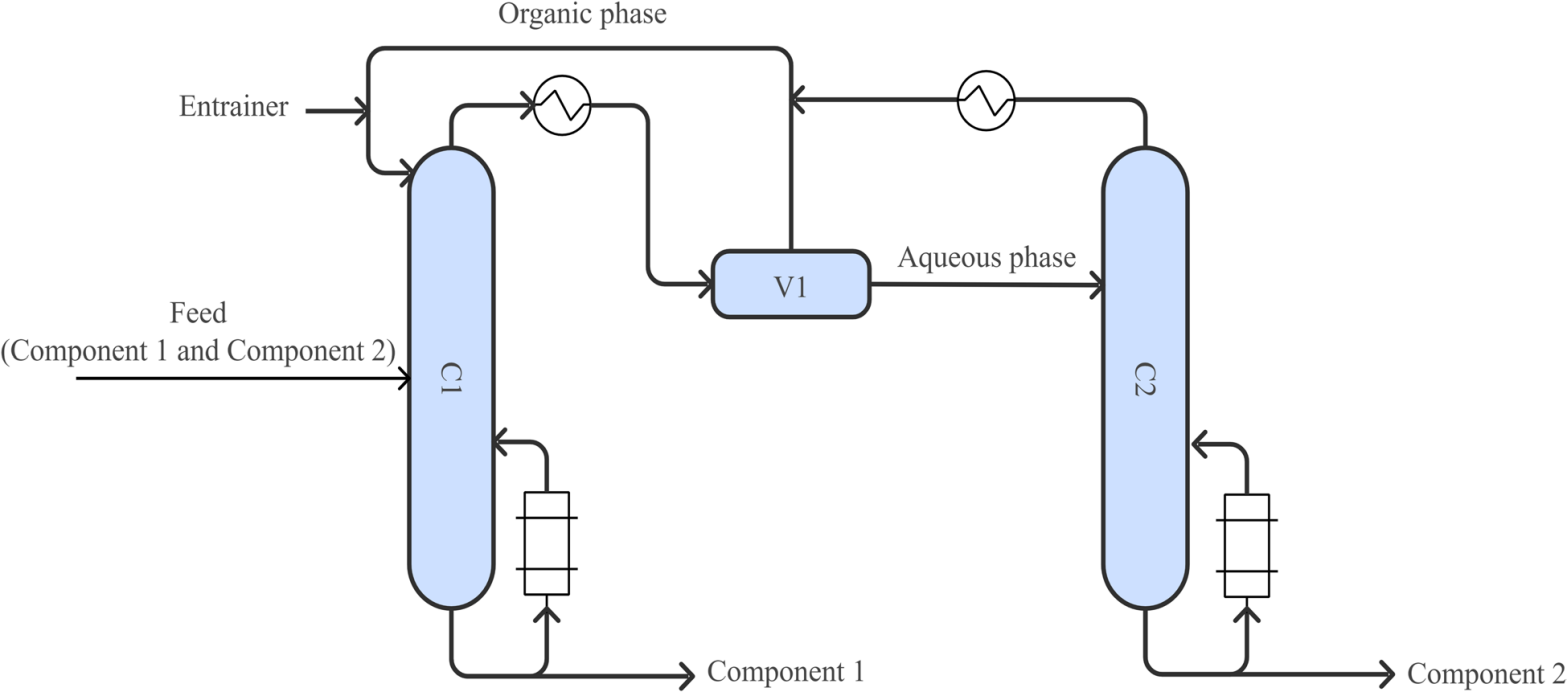
Conventional azeotropic distillation process. (C1) Azeotropic distillation column; (C2) Entrainer recovery column; (V1) Phase splitter

Azeotropic distillation (Fig. 7) involves adding an entrainer to alter the relative volatilities and break the azeotropic composition. Based on the miscibility between the entrainer and the system components, azeotropic distillation is classified into homogeneous and heterogeneous types. Heterogeneous azeotropic distillation is more commonly applied industrially due to its ease of phase separation and entrainer recovery (Janakey Devi et al. 2017b). In isopropanol dehydration, entrainers such as n-propyl acetate, isopropyl acetate (Janakey Devi et al. 2017a), and isooctane (De Guido et al. 2021) have shown promising results. For n-propanol, agents like n-propyl acetate (Janakey Devi et al. 2017b) and diisopropyl ether (Pla-Franco et al. 2019) have achieved recovery rates exceeding 99.0%. However, these methods often require large quantities of entrainers, increasing the complexity and cost of recovery.

**Fig. 8:**
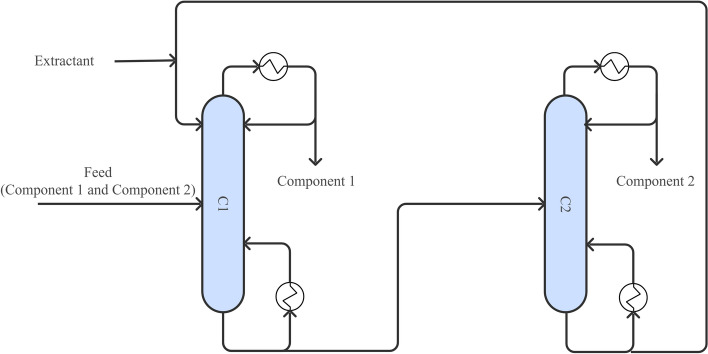
Conventional extractive distillation process. (C1) Extractive distillation column; (C2) Solvent recovery column

Extractive distillation (Fig. 8) utilizes a selective solvent to preferentially extract the target component from the azeotropic mixture (Wu et al. 2020b). Typical solvents include butanol, glycerol, ethyl acetate, and butyl propionate. For example, isobutyl acetate, butyl acetate, and butyl propionate are recognized as effective extractants for isopropanol (Liu et al. 2018), while butyl butyrate and n-pentyl acetate are efficient in separating low-concentration propanol solutions (Peng et al. 2020). Glycerol has also been shown to achieve 99.9% purity in propanol-water separations (Wang et al. 2021a; Wu et al. 2020b). In recent years, ionic liquids (ILs) have emerged as advanced extractants due to their low volatility, thermal stability, and ease of recovery (An et al. 2015; Dai et al. 2015). Ma et al. (2019) demonstrated the effectiveness of 1-ethyl-3-methylimidazolium dicyanamide in isopropanol–water separation, while (Li et al. 2022) identified 1-octyl-3-methylimidazolium acetate as the most efficient IL for separating benzene and n-propanol.

**Fig. 9:**
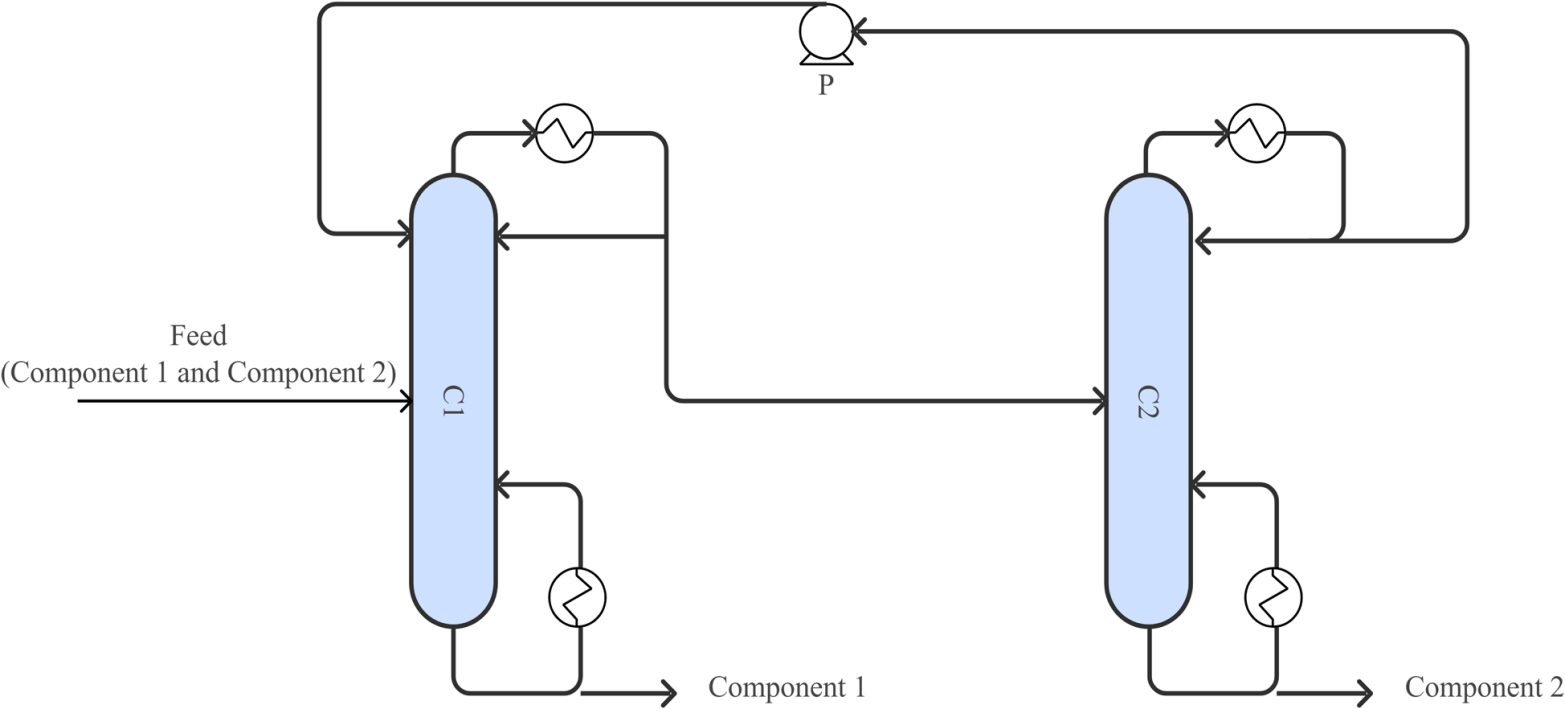
Conventional pressure swing distillation process. (C1) Pressurized column; (C2) Atmospheric column; (P) Booster pump

Pressure-swing distillation (Fig. 9) leverages the pressure-dependent variation in azeotropic composition to achieve separation without the need for additional entrainers (Cui et al. 2020; Wang et al. 2016). This technique is particularly effective for separating ternary azeotropic systems, such as n-propanol/acetonitrile/isopropanol (Yu et al. 2023b), n-propanol/water/tetrahydrofuran (Yu et al. 2023a), and diisopropyl ether/isopropanol/water (Hazelwood et al. 2008). By fine-tuning the operating pressure, pressure-swing distillation can enhance separation efficiency and lower energy consumption.

Despite their promising results, these advanced distillation techniques remain primarily confined to laboratory-scale applications due to engineering complexity, the cost-intensive nature of solvent recovery, and challenges in process control. More critically, distillation is an energy-intensive process (Xue et al. 2025). Therefore, reducing the energy footprint of distillation or developing low-carbon alternative separation technologies is imperative for achieving economically and environmentally sustainable propanol production.

### Membrane separation

Membrane separation technology has emerged as an efficient and energy-saving technique, demonstrating significant advantages in the separation of propanol (Wu et al. 2020a). Standard membrane separation techniques include pervaporation (PV) and vapor permeation (VP). PV utilizes the selective permeability of a membrane to separate components in a liquid mixture, driven by a vapor pressure gradient across the membrane (Moriyama et al. 2018; Vane 2019). Compared to conventional azeotropic distillation, PV provides higher separation selectivity, reduced energy consumption, and greater flexibility in process design (Kwon et al. 2018). VP operates on a similar principle to PV but differs in that its feed is in the gas phase, thereby eliminating the latent heat consumption required for liquid vaporization (Vane 2020). Furthermore, membrane swelling is significantly reduced in the gas phase, which contributes to the longer operational lifespan of VP membranes in industrial applications (Harvianto et al. 2016). In diol-water systems, VP typically exhibits a higher separation factor but a relatively lower flux than PV, and accordingly receives more attention.

Polyvinyl alcohol (PVA) has been extensively investigated as a membrane material for pervaporation-based propanol dehydration due to its excellent hydrophilicity and film-forming capability (Wang et al. 2023a). However, it still suffers from water-induced swelling and limited thermal stability under practical operating conditions (Yang et al. 2020a). To enhance membrane performance, recent studies have focused on developing mixed matrix membranes (MMMs) by incorporating inorganic fillers into the PVA matrix to improve its structural integrity and separation performance. Reported fillers include zeolites (Huang et al. 2019; Wang et al. 2023a), metal–organic frameworks (MOFs) (Lin et al. 2021a; Wang et al. 2023c), silica nanoparticles (Reino Olegário da Silva et al. 2020), and other inorganic particles (Wang et al. 2018b), all of which have demonstrated significant improvements in membrane separation efficiency and permeation flux. In propanol dehydration research, PVA-based MMMs incorporating zeolites have been widely adopted. For example, membranes fabricated by incorporating ZSM-5 zeolite into PVA have shown effective dehydration of n-propanol, with significantly enhanced separation performance compared to pristine PVA membranes. At 20 wt% zeolite loading and an operating temperature of 60 °C, the composite membrane achieved a separation factor of 820 and a total permeation flux of 825 g/m^2^·h in an 80 wt% aqueous propanol solution (Wang et al. 2023b). PVA-based MMMs loaded with H-β zeolite have also exhibited excellent performance in the separation of ethanol, n-propanol, and isopropanol, with their high separation efficiency primarily attributed to the synergistic effect of the molecular sieve structure and hydrophilic nature of the zeolite. A membrane loaded with 20 wt% zeolite has demonstrated stable operation for over 144 h at 60 °C. For a feed containing 10 wt% water, the corresponding separation factors are 1204 for isopropanol, 1391 for n-propanol, and 1645 for ethanol, with permeation fluxes of 719, 702, and 684 g/m^2^·h, respectively (Wang et al. 2023a). Ag–NaZ zeolite particles synthesized via ion exchange and embedded into crosslinked PVA membranes have also demonstrated effectiveness in isopropanol–water dehydration, achieving a separation factor of up to 2717 for a 9 wt% isopropanol feed at 40.8 °C (Kwon et al. 2018).

Although membrane separation technology presents numerous advantages for propanol dehydration, its large-scale industrial application remains limited by several critical challenges. Among these, membrane fouling and decline in permeation flux are the primary factors limiting long-term stable operation. In addition, the high polarity of propanol can induce swelling in hydrophilic membranes such as PVA, thereby disrupting the membrane's internal cross-linked structure (Bowen et al. 2004). Economic constraints represent another significant barrier to the large-scale deployment of membrane separation technology, necessitating the integration of hybrid processes (e.g., membrane separation-distillation) or optimization of operational parameters (e.g., lower operating pressure) to improve overall cost-effectiveness.

### Membrane distillation

Membrane distillation is a hybrid separation process that integrates membrane separation and distillation principles, and has witnessed rapid development in recent years (Lone et al. 2015). Although membrane separation theoretically offers up to 90% energy savings compared to conventional distillation columns (Wang et al. 2020), its full-scale replacement remains impractical due to current technological and economic limitations. The integration of membrane separation with other separation technologies has become a key focus of current research.

Membrane distillation achieves separation through the selective vapor transport across hydrophobic membranes, offering advantages such as simple configuration, low energy consumption, and no requirement for entrainers (Sommer and Melin 2004). The separation efficiency of membrane distillation primarily depends on the diffusion rate and adsorption behavior of solutes within the membrane material. In the context of process integration, a distillation-pervaporation hybrid system employing glycerol as an extractant was developed to effectively separate a ternary mixture of *n*-propanol, acetonitrile, and water (Wang et al. 2021a). Further validation in an *n*-propanol-water system confirmed that the integrated process can achieve a permeation flux ranging from 304 to 1216 g/(m^2^·h), with product purity reaching 99.9% (Toth 2022). Additionally, optimization of membrane module dimensions and heating strategies significantly reduced the annual total cost (Do Thi and Toth 2025). From an economic perspective, the distillation–vapor permeation hybrid process has been shown to reduce total operating costs by approximately 77% compared to conventional azeotropic distillation (Harvianto et al. 2016). Furthermore, combining pervaporation with extractive distillation has been reported to reduce the annual comprehensive cost of *n*-propanol dehydration by more than 20%, indicating strong techno-economic viability (Wu et al. 2020b).

### Gas stripping

**Fig. 10:**
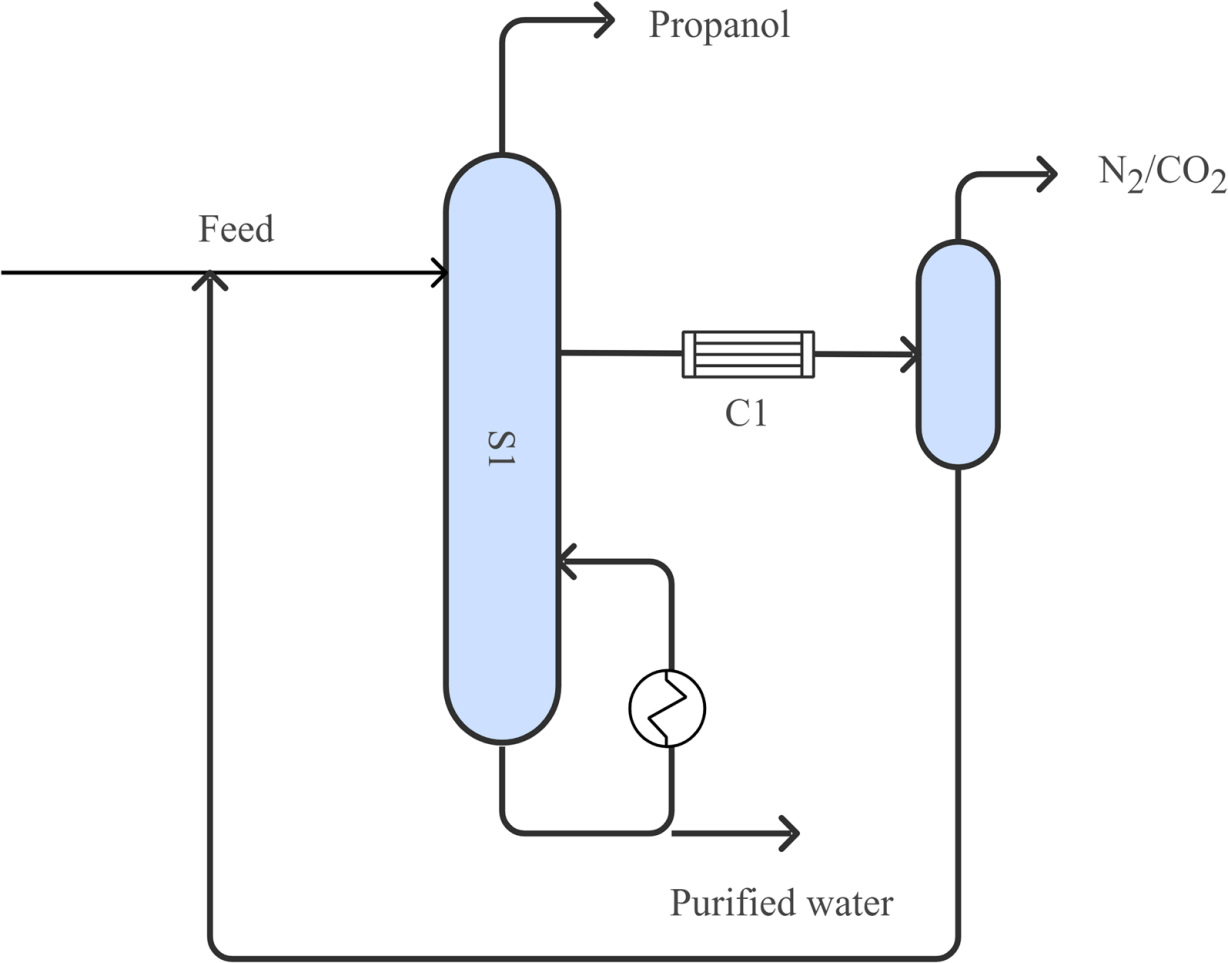
Conventional gas stripping process. (S1) Stripping tower; (C1) Condenser

Gas stripping (Fig. 10) involves introducing inert gases (e.g., N₂, CO₂) into fermentation broth to selectively remove volatile metabolites such as propanol (Ezeji et al. 2004; Pyrgakis et al. 2016). Integrating gas stripping into fermentation effectively reduces propanol concentration in the broth, alleviating product inhibition and significantly enhancing fermentation efficiency (de Vrije et al. 2013; Pyrgakis et al. 2016). Studies have shown that genetically engineered strains utilizing gas stripping can achieve glucose consumption (132.9 g/L) and produce 35.6 g/L IBE mixtures (Lee et al. 2012). Recovery of over 99% of the IBE mixtures using gas stripping has also been demonstrated (Pyrgakis et al. 2016). In fed-batch fermentation, gas stripping has significantly enhanced isopropanol production. (Inokuma et al. 2010) reported that using gas stripping increased isopropanol titers to 143 g/L (2378 mM), more than tripling the yield compared to traditional shake-flask fermentation. Further improvement was observed with an optimized genetically engineered strain, which enabled a 97.68% isopropanol recovery rate (10.01 ± 0.95 g/L) over 100 h of fermentation. Glucose consumption reached 205 g, with a conversion efficiency of 0.34 mol/mol(Ko et al. 2022a). Minimal byproduct formation was observed, with lactic acid, acetic acid, and succinic acid all below 1.0 g/L, indicating high product selectivity.

Consequently, various emerging separation technologies exhibit considerable potential for application in propanol purification. The need for future research is urgent, as it should focus on optimizing process parameters, reducing energy and operational costs, and developing advanced separation materials to further enhance both separation efficiency and economic viability. Moreover, the integration of multiple separation techniques is anticipated to become a key research focus, potentially enabling significant breakthroughs in the field of propanol separation.

## Key challenges and prospects

Although significant progress has been made in microbial fermentation for the biosynthesis of bio-propanol, its industrial application remains challenged by several key factors (Hocq and Sauer 2022), which are primarily summarized as follows.

### Feasibility and metabolic efficiency limitations of industrial strains

Currently, natural isopropanol-producing strains, such as *Clostridium beijerinckii* (Vieira et al. 2021; Yao et al. 2022) and *Clostridium acetobutylicum* (Hijosa-Valsero et al. 2019), exhibit an inherent capacity for alcohol synthesis, but still require extensive metabolic engineering to overcome limitations in yield and tolerance (Zhou et al. 2019). In contrast, natural n-propanol-producing strains, including *Propionibacterium acidipropionici* (Dishisha et al. 2012), Yeast (Díaz-Montaño et al. 2008), and *Lactobacillus* (Djameh et al. 2019), generally suffer from low production titers and conversion efficiencies, which fall short of industrial requirements. Numerous studies have employed genetic engineering strategies to enhance microbial productivity; however, these engineered strains often still face challenges such as limited microbial robustness, imbalanced metabolic flux distribution (Lin et al. 2021b; Shen and Liao 2008), and constraints in enzyme activity or substrate range (Ohtake et al. 2022).

Therefore, future research should focus on three key directions: (i) discovery and characterization of novel microbial resources, particularly those with high tolerance to propanol or complete biosynthetic pathways; (ii) optimization of fermentation conditions to identify the most suitable growth environments and cultivation strategies for high-efficiency production; and (iii) exploration of co-culture strategies that exploit metabolic complementarity between strains to enhance carbon flux efficiency and product specificity. For instance, co-fermentation systems involving *Clostridium* and *Propionibacterium* (Hocq and Sauer 2022) or *Lactobacillus* and Yeast (Djameh et al. 2019) have demonstrated improved yields and selectivity, offering promising prospects for stable and efficient microbial production platforms.

### Underutilized potential of biomass waste resources

The current fermentation-based synthesis of bio-propanol heavily relies on expensive carbon sources, such as glucose (Cui et al. 2020; Ferreira dos Santos Vieira et al. 2022) and glycerol (Dishisha et al. 2012), which limits both economic viability and sustainability. In contrast, low-value biomass wastes (including food waste and agricultural residues) are widely available and economically attractive as alternative feedstocks. Studies have shown that agro-industrial byproducts, such as coffee silver skin (Procentese et al. 2018), tomato waste (Hijosa-Valsero et al. 2019), and sugarcane bagasse (Vieira et al. 2021), can be effectively utilized for the microbial production of isopropanol and n-propanol. However, practical implementation remains constrained by the need for energy-intensive and chemically demanding pretreatment and saccharification processes (Vieira et al. 2021; Xin et al. 2017). Moving forward, emphasis should be placed on identifying widely accessible biomass waste streams that require minimal preprocessing, such as municipal food waste, crop straw, and fruit and vegetable discards. These feedstocks should be coupled with compatible microbial consortia and mild enzymatic pretreatment methods to establish low-cost and efficient systems for propanol production.

### Technical contradictions between separation efficiency and energy demand

Efficient separation of propanol remains a significant bottleneck for its industrial-scale production. Due to its strong polarity and propensity to form azeotropes with water (Kwon et al. 2018; Wang et al. 2023a), conventional distillation methods are energy-intensive and thermodynamically unfavorable (Xue et al. 2025). Although advanced separation techniques, such as membrane separation, membrane distillation, and gas stripping, have demonstrated promising results under laboratory conditions, their scalability is hindered by challenges related to complex broth handling, operational stability, and energy consumption (Choi Yong et al. 2014; Inokuma et al. 2010). Future development should draw from advances in modern chemical process engineering, including membrane-integrated systems, intelligent process control, energy recovery, and process intensification strategies. Such innovations are crucial for developing integrated fermentation-separation platforms that enhance overall process efficiency, cost-effectiveness, and environmental sustainability.

## Conclusions

This review summarizes recent advances in microbial fermentation for the production of bio-propanol. Topics include microbial strain development, pathway design, and downstream separation technologies. As a renewable and environmentally friendly fuel, bio-propanol has strong industrial potential. However, several key challenges remain. Firstly, there is a lack of robust and efficient microbial strains for the biosynthesis of propanol. Current strains used for isopropanol and n-propanol production exhibit significant limitations in tolerance. These issues hinder high-yield and cost-effective fermentation. Secondly, the fermentation process remains heavily reliant on high-purity carbon sources, and low-cost biomass alternatives are underused. This reliance severely constrains both the economic feasibility and sustainability of bio-propanol production. Finally, the separation and purification of propanol present technical barriers. Conventional distillation is energy-intensive and operationally complex. Novel separation methods, such as membrane technologies, have shown potential in the laboratory. However, their industrial scalability, stability, and cost control remain unresolved.

To address these issues, future research should establish diversified microbial fermentation platforms. Efforts should be directed toward mining natural microbial resources and integrating synthetic biology and metabolic engineering approaches to construct functional and high-performance engineered strains. Co-culture systems should be explored to harness metabolic complementarity and achieve synergistic interactions among microbial consortia, thereby improving overall propanol synthesis efficiency. In parallel, the efficient utilization of low-value biomass should be promoted by developing simplified and energy-efficient pretreatment and saccharification processes, thereby reducing feedstock costs and improving carbon source conversion. Furthermore, the integration of upstream fermentation with downstream separation processes should be strengthened, and the development of low-energy, high-selectivity separation technologies is crucial to improving the environmental sustainability and economic viability of the overall production process.

## Data Availability

Not applicable.

## Abbreviations

ABE: Acetone-butanol-ethanol
IBE: Isopropanol-butanol-ethanol
PV: Pervaporation
VP: Vapor permeation
PVA: Polyvinyl alcohol
MMMs: Mixed matrix membranes
